# SIRT2 promotes the viability, invasion and metastasis of osteosarcoma cells by inhibiting the degradation of Snail

**DOI:** 10.1038/s41419-022-05388-2

**Published:** 2022-11-07

**Authors:** Yonghao Tian, Ruijuan Liu, Xiaoyan Hou, Zhixiao Gao, Xinyu Liu, Weifang Zhang

**Affiliations:** 1 Department of Orthopedic Surgery, Qilu Hospital of Shandong University, Jinan, Shandong China; 2 Department of Microbiology, School of Basic Medical Sciences, Cheeloo College of Medicine, Shandong University, Jinan, Shandong China

**Keywords:** Bone cancer, Cell invasion, Bone metastases

## Abstract

Osteosarcomas (OS) are highly metastatic and usually lead to poor outcomes. Epithelial-mesenchymal transition (EMT) is reported to be a critical event in metastasis. SIRT2 exerts dual functions in many different tumors. However, the underlying molecular mechanisms of SIRT2 in osteosarcoma cell metastasis and the question of whether SIRT2 regulates EMT have not been fully explored. In this study, we confirmed that SIRT2 was highly-expressed in human osteosarcoma MG63 and Saos-2 cell lines. The viability, migration and invasion of osteosarcoma cells were inhibited by knockdown of SIRT2 and were enhanced by overexpression of SIRT2. Moreover, SIRT2 positively regulated EMT and upregulated the protein levels of the mesenchymal markers N-cadherin and Vimentin and the levels of MMP2 and MMP9. A xenograft mouse model showed that SIRT2 knockdown in osteosarcoma cells led to reduced tumor growth, decreased expression of mesenchymal markers and impaired lung and liver metastasis in vivo. Furthermore, we showed that SIRT2 interacted with and upregulated the protein level of the EMT-associated transcription factor Snail. SIRT2 inhibited Snail degradation via its deacetylase activity. Knockdown of Snail abrogated the promoting effects of SIRT2 on migration and invasion of osteosarcoma cells. In conclusion, SIRT2 plays a crucial role in osteosarcoma metastasis by inhibiting Snail degradation and may serve as a novel therapeutic target to manage osteosarcoma.

## Introduction

Osteosarcoma (OS) is the most common primary malignant bone tumor, and is mainly composed of osteoid and cartilaginous matrix as well as fibrous tissues [1]. The annual incidence of osteosarcoma is 3 to 4 cases per million [2]. The incidence in adolescents aged 15-19 is the highest, and compared with females (10.7 per million), males have a higher incidence (19.3 per million), which suggests that males are more likely to be affected by osteosarcoma [3]. Although osteosarcoma can occur in any bone, the metaphysis of long bones is the most common site [4]. Osteosarcoma is highly aggressive, and it can quickly invade surrounding tissues and disseminate through the body. Due to its strong metastasis, osteosarcoma is associated with a high mortality rate, with a 5-year survival rate of approximately 50–60% [5]. Tumor recurrence, high lung metastasis and chemotherapy resistance are the main reasons for the poor prognosis [5]. Therefore, it is essential to explore the molecular mechanism of metastasis and develop new strategies for the treatment of osteosarcoma.

Epithelial-mesenchymal transition (EMT) plays a key role in wound healing, embryonic development and tumor metastasis [6]. During the progression of EMT, cell-cell junctions and apico-basal polarity are lost, and invasive properties are acquired. EMT transforms early tumors into aggressive malignant tumors [7]. During cancer progression, tumor cells that undergo EMT fall off and enter lymphatic and blood vessels, causing the systemic spread and development of secondary tumors in distant organs [8]. EMT is the key step in metastatic malignant tumors of epithelial origin. In addition, mounting evidence has proven the crucial role of EMT in tumors of mesenchymal origin, such as osteosarcoma [9, 10].

Sirtuins (silent information regulators) are NAD^+^ (nicotinamide adenine dinucleotide)-dependent type III histone deacetylases (HDACs), which include a family of proteins with homology to silent information regulator 2 (Sir2) in Saccharomyces cerevisiae [11]. The seven sirtuin family members (SIRT1-SIRT7) show diversity in subcellular localization and function. SIRT1, SIRT6 and SIRT7 are mainly located in the nucleus, and SIRT3, SIRT4 and SIRT5 are located in the mitochondria, while SIRT2 is the only sirtuin mainly located in the cytoplasm [12]. The N-terminus of SIRT2 has a leucine-rich nuclear export signal (NES), which can regulate its nucleoplasmic localization [13]. SIRT2 levels increase during mitosis and accumulate in the nucleus when treated with a nuclear export inhibitor, and overexpression of SIRT2 markedly prolongs the mitotic phase [14, 15]. Mechanistically, SIRT2 is localized on chromatin and deacetylates histone H4K16Ac, which is vital for chromatin condensation during mitotic phase [16]. Thus, SIRT2 can instantly migrate to the nucleus during the transition period of G2/M and is involved in the regulation of mitosis. Current research shows that SIRT2 plays an important role in many physiological and pathological processes, such as proliferation, the cell cycle, apoptosis, genome integrity, cell metabolism, infection and inflammation. In different tumor types, SIRT2 may act as an activator or inhibitor [17]. There are many studies on SIRT2 in breast cancer, liver cancer, lung cancer, leukemia and other malignant tumors, but no relevant research on its role in the development of osteosarcoma has been reported.

In this study, we identified the oncogenic role of SIRT2 in osteosarcoma and explored the underlying molecular mechanism of SIRT2 in the invasion and metastasis of osteosarcoma cells through in vivo and in vitro experiments. Importantly, we report that SIRT2 promoted the invasion and metastasis by inhibiting the degradation of the transcription factor Snail via its deacetylase activity. Therefore, SIRT2 might be a promising candidate for use against the metastasis of human osteosarcoma.

## Results

### SIRT2 is upregulated and promotes the viability, migration and invasion of osteosarcoma cells

**Fig. 1:**
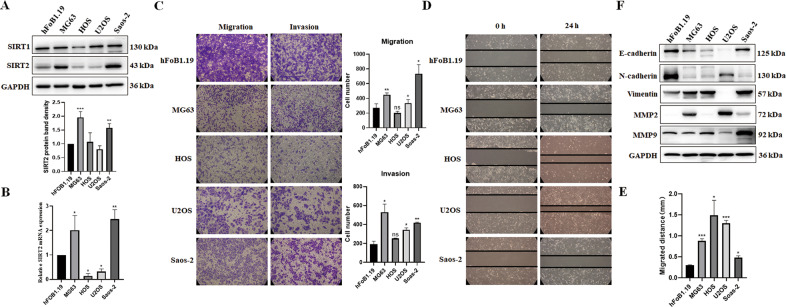
SIRT2 is elevated in osteosarcoma cells. **A** Western blot was used to detect the protein expression levels of SIRT1 and SIRT2 in hFOB1.19 osteoblasts and osteosarcoma cell lines MG63, HOS, U2OS and Saos-2. Data from a representative experiment of at least three independent samples are shown. A grayscale protein histogram is shown below. ImageJ software was used for quantitative analysis of protein band density. First, the bands of the target protein were framed with the same rectangle to obtain the gray value of each band. Second, the gray values of the loading control were acquired in the same way. Finally, the gray value of the target protein was divided by the gray value of the corresponding loading control to obtain a relative value for each band and the relative value of the first band in each group was set as “1”. **B** qRT-PCR was employed to detect the mRNA expression level of SIRT2 in different osterosarcoma cells and control cells. **C** Transwell assays were used to analyze the migration and invasion of different osteosarcoma cells and control cells. Results from three experiments are summarized in a histogram format. **D** A wound-healing experiment was used to analyze the migration of different osteosarcoma cells and control cells. **E** Results from three experiments are summarized in a histogram format. **F** The expression of EMT-related proteins in osteosarcoma cells and control cells were detected by Western blot. Results representative of three experiments are shown. (*P < 0.05, **P < 0.01, ***P < 0.001).

SIRT1, SIRT2, SIRT6 and SIRT7 proteins are mainly localized in the nucleus or cytoplasm and are more likely to play important roles in the development of osteosarcoma. Previous reports have shown that SIRT6 and SIRT7 proteins were elevated in osteosarcoma cells, and they promoted the migration and invasion of osteosarcoma cells by different mechanisms [18, 19]. Thus, we focused on SIRT1 and SIRT2 in this study and detected the expression of these two proteins in hFOB1.19 (human osteoblasts) and several osteosarcoma cell lines MG63, HOS, U2OS and Saos-2. The protein level of SIRT2 was significantly upregulated in MG63 and Saos-2 cells compared with human osteoblasts (Fig. 1A). However, the expression of SIRT1 protein did not change greatly in osteosarcoma cells, which suggests that SIRT2 may play a more important role than SIRT1 in the development of osteosarcoma. The mRNA level of SIRT2 was also significantly increased in osteosarcoma MG63 and Saos-2 cells (Fig. 1B). Transwell assay showed that in addition to MG63 and Saos-2 cells, U2OS cells also had increased cell migration and invasion compared with hFOB1.19 control cells (Fig. 1C). The wound-healing experiment showed that the migrating speed of cells to the scratch was significantly increased in all four osteosarcoma cell lines (Fig. 1D, E). This may be because the proliferation rate of hFOB1.19 cells is the slowest and the migrating speed depends largely on the proliferation rate of cells [20]. Since osteosarcoma is a malignant stromal tumor, the expressions of epithelial and mesenchymal proteins were detected. The epithelial marker E-cadherin was decreased in all osteosarcoma cell lines compared with hFOB1.19 cells (Fig. 1F). The expression of mesenchymal marker Vimentin and the matrix metalloproteinase 2 (MMP-2) which degrades and remodels the extracellular matrix (ECM) was increased in most of the osteosarcoma cell lines. However, an obvious increase of MMP-9 was only observed in Saos-2 cells. Unexpectedly, the mesenchymal marker N-cadherin was decreased in all osteosarcoma cell lines. Our data showed that not all the mesenchymal proteins and MMP proteins were increased in MG63 and Saos-2 cells that expressed high level of SIRT2. We suppose that there might be other proteins that regulate the expression of N-cadherin and MMP9 in these cells.

**Fig. 2:**
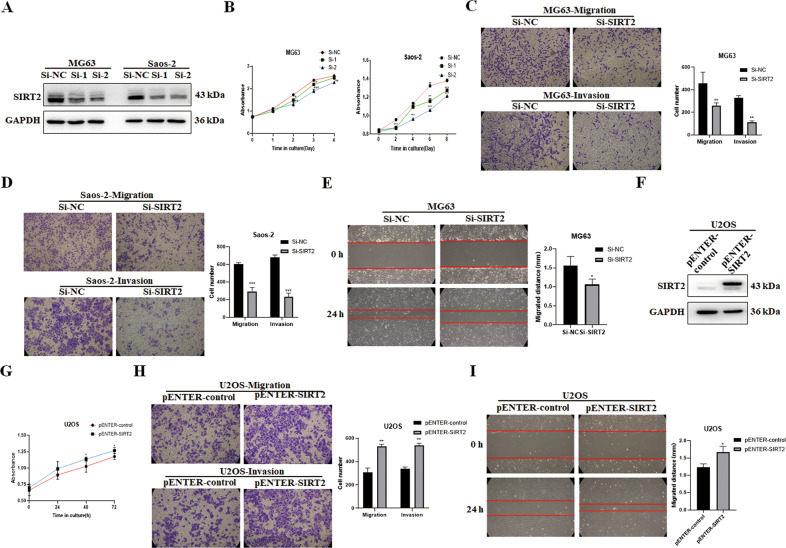
SIRT2 promotes migration and invasion of osteosarcoma cells. **A** Two SIRT2 siRNAs were transfected into the osteosarcoma cell lines MG63 and Saos-2, and Western blot was used to detect the knockdown efficiency of SIRT2. **B** A CCK-8 assay was used to detect cell viability in cells with SIRT2 knockdown for 4 or 8 consecutive days. **C**, **D** Transwell assays were employed to study the effect of SIRT2 knockdown (Si-2) on cell migration and invasion ability. Results from three experiments are summarized in a histogram format. **E** The photograph of the wound-healing experiment showed the migration distance of osteosarcoma cells to the scratch after SIRT2 knockdown (Si-2) at 0 h and 24 h. Results from three experiments are summarized in a histogram format. **F** The expression of SIRT2 was detected after transfection of the SIRT2 plasmid. **G**–**I** The CCK-8 assay, Transwell assay and wound-healing experiment were used to analyze SIRT2 overexpression on cell viability, migration and invasion. Results from three experiments are summarized in a histogram format. (*P < 0.05, **P < 0.01, ***P < 0.001).

Since SIRT2 is highly expressed in osteosarcoma cell lines MG63 and Saos-2, two small interfering RNAs (siRNAs) of SIRT2, Si-SIRT2-1 (abbreviated as Si-1) and Si-SIRT2-2 (abbreviated as Si-2) were used to transfect MG63 and Saos-2 cells respectively (Fig. 2A). The CCK-8 assay showed that SIRT2 knockdown inhibited the viability of osteosarcoma cells (Fig. 2B). The results of Transwell assay showed that the number of cells passing through the chamber was decreased after SIRT2 knockdown, indicating that SIRT2 knockdown inhibited the migration and invasion of osteosarcoma cells (Fig. 2C, D). The results of the wound-healing experiment showed that the migrating speed to the scratch was reduced after SIRT2 knockdown (Fig. 2E). The expression level of SIRT2 in osteosarcoma U2OS cells was relatively low, so the pENTER-SIRT2-c-Flag-His plasmid was transfected into U2OS cells (Fig. 2F). Our results showed that the overexpression of SIRT2 greatly increased the viability, migration and invasion of osteosarcoma cells (Fig. 2G–I). In summary, SIRT2 is increased in osteosarcoma cells and promotes the viability, migration and invasion of osteosarcoma cells.

### SIRT2 promotes EMT progression

**Fig. 3:**
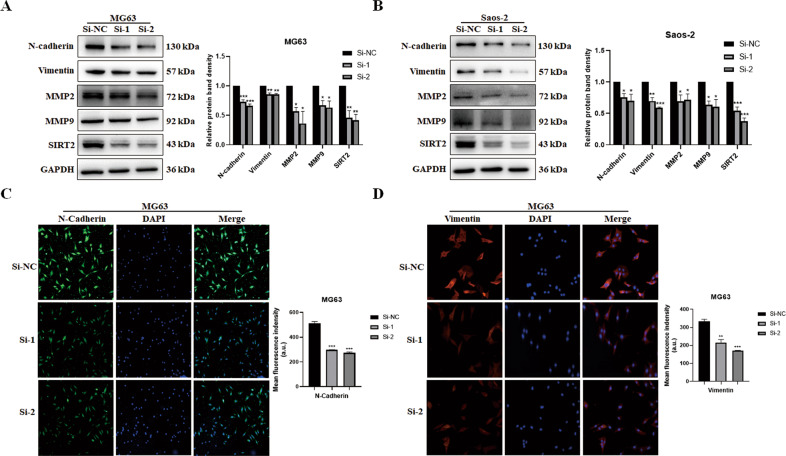
SIRT2 knockdown downregulates the expression of mesenchymal proteins. **A**, **B** The expression of EMT-related proteins was detected by Western blot after SIRT2 knockdown in MG63 and Saos-2 cells. Results from three experiments are summarized in a histogram format. **C**, **D** Representative immunofluorescence of N-cadherin and Vimentin after SIRT2 knockdown was shown. The right panel was the summarized data of immunofluorescence score of N-cadherin and Vimentin expression density. (*P < 0.05, **P < 0.01, ***P < 0.001).

**Fig. 4:**
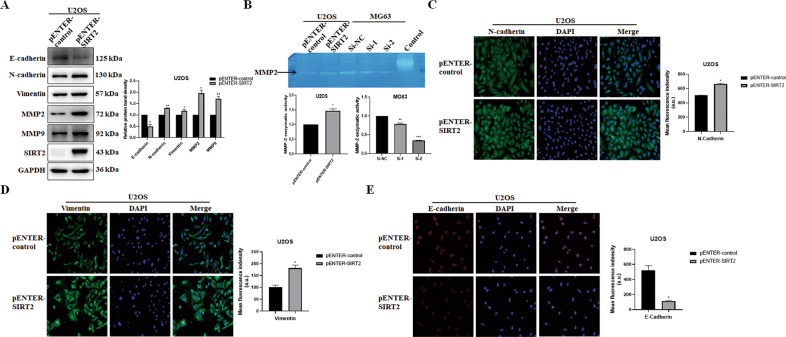
SIRT2 overexpression downregulates the epithelial marker and upregulates mesenchymal proteins. **A** The expression of EMT-related proteins was detected by Western blot after SIRT2 overexpression in U2OS cells. Results from three experiments are summarized in a histogram format. **B** The gelatin zymogram assay was used to detect the activity of MMPs with SIRT2 overexpression or knockdown. Data from a representative experiment of three independent samples are shown. **C**–**E** Representative immunofluorescence of N-cadherin, Vimentin and E-cadherin with SIRT2 overexpression was shown. The right panel was the summarized data of immunofluorescence score of related protein. (*P < 0.05, **P < 0.01, ***P < 0.001).

EMT is an important way to promote cell invasion and metastasis. We then detected the expression of EMT-related proteins after SIRT2 knockdown in MG63 and Saos-2 cells. The results showed that the expression levels of the mesenchymal markers N-cadherin and Vimentin were decreased after SIRT2 knockdown; MMP-2 and MMP-9 were also decreased (Fig. 3A, B). The expression of N-cadherin and Vimentin was further detected by immunofluorescence. The intensity of immunofluorescence of N-cadherin and Vimentin was significantly weakened after SIRT2 knockdown (Fig. 3C, D). N-cadherin was distributed in the nucleus and cytoplasm, while Vimentin was mainly distributed in the cytoplasm. SIRT2 overexpression downregulated the expression of the epithelial marker E-cadherin and upregulated the expression of N-cadherin, Vimentin, MMP2, and MMP9 in U2OS cells (Fig. 4A). The gelatin zymogram assay was used to detect the activity of MMPs. SIRT2 overexpression significantly increased the enzymatic activity of MMP2, while SIRT2 knockdown decreased the enzymatic activity of MMP2 (Fig. 4B). The immunofluorescence intensity of N-cadherin and Vimentin was notably increased with SIRT2 overexpression (Fig. 4C, D). In contrast, SIRT2 overexpression significantly reduced the immunofluorescence intensity of E-cadherin (Fig. 4E). Thus, SIRT2 promotes EMT by upregulating the mesenchymal proteins and downregulating the epithelial marker.

### SIRT2 promotes tumor growth and invasion in vivo

**Fig. 5:**
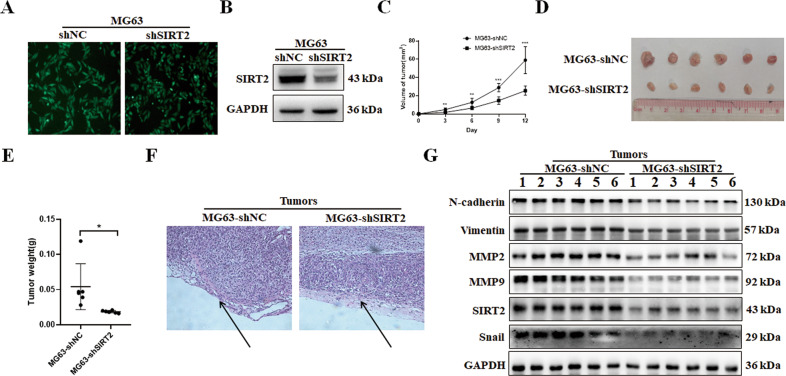
SIRT2 knockdown inhibited the tumorigenic ability of osteosarcoma cells. **A** The efficiency of SIRT2 shRNA lentivirus infection in MG63 cells was observed under a fluorescence microscope. **B** Western blot was used to detect SIRT2 expression after SIRT2 shRNA infection of MG63 cells. **C** The tumor growth curve of nude mice subcutaneously injected with MG63-shSIRT2 and control cells. **D** The tumors of the nude mice in the two groups were isolated and photographed. **E** The tumors in the nude mice were weighed and compared. **F** H&E staining of tumor tissues. Data from one pair of representative staining of 6 tumor tissues are shown. **G** The EMT-related proteins were detected in the tumors of the two groups. Data from one representative experiment of three are shown. (*P < 0.05, **P < 0.01, ***P < 0.001).

We infected MG63 cells with SIRT2 shRNA lentivirus and established an osteosarcoma cell line with stable SIRT2 knockdown (MG63-shSIRT2) (Fig. 5A, B). MG63-shSIRT2 cells or control cells were injected subcutaneously into the left forelimb of nude mice. The tumor sizes in the nude mice were observed and compared, and the tumor volumes were measured regularly to record the tumor growth curves (Fig. 5C). It was shown that SIRT2 knockdown significantly inhibited tumor growth. The mice were sacrificed 12 days after injection, and the tumors were isolated, photographed and weighed. The results showed that the volume and weight of tumors in the SIRT2 knockdown group were significantly lower than those in the control group (Fig. 5D, E), indicating that SIRT2 knockdown inhibited the proliferation of osteosarcoma cells in nude mice. H&E staining of tumor tissues showed that SIRT2 knockdown group had a complete envelope of tumors, while the tumor envelope was disrupted in the control group, indicating that SIRT2 knockdown inhibited tumor invasiveness (Fig. 5F). The expression levels of EMT-related proteins in tumors of the SIRT2 knockdown group and control group were detected (Fig. 5G). N-cadherin, Vimentin, MMP2, and MMP9 were all decreased in the tumors of the SIRT2 knockdown group.

**Fig. 6:**
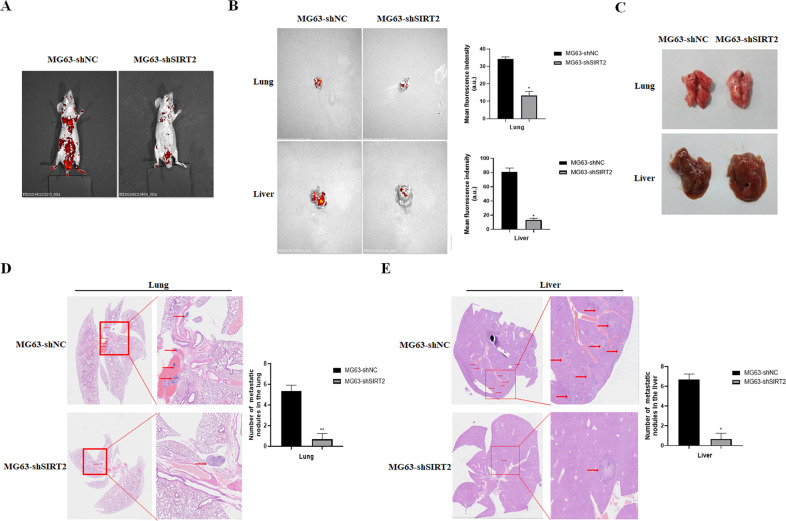
SIRT2 knockdown inhibited the metastatic ability of osteosarcoma cells in vivo. **A** Small animal in vivo imager was used to detect the metastasis of osteosarcoma cells in the SIRT2 knockdown group and the control group. **B** Fluorescence images of the lung and liver in the SIRT2 knockdown group and control group. A representative fluorescence image of at least three tissue samples is shown. The right panel is the summarized data of immunofluorescence density. **C** Photographs of the lung and liver of the two groups of nude mice. **D**, **E** H&E staining of paraffin-embedded lung and liver sections. The right panel is a magnification of the rectangle in the left panel, with arrows showing metastatic nodules. Number of metastatic nodules in the lung or liver is summarized in a histogram format. (*P < 0.05, **P < 0.01).

To explore the effect of SIRT2 on the metastatic ability of osteosarcoma cells in vivo, MG63-shSIRT2 or control cells were injected into the tail vein of nude mice. Our results showed that the fluorescence intensity of nude mice in the control group was obviously higher than that in the SIRT2 knockdown group, and the fluorescence was mainly distributed in the chest and abdomen (Fig. 6A). Importantly, in mice xenografted with MG63-shSIRT2 cells, the fluorescence intensity of the lung and liver was attenuated, and metastatic nodules were greatly reduced (Fig. 6B, C). H&E staining further confirmed that the SIRT2 knockdown group had significantly fewer metastatic nodules in the lung and liver (Fig. 6D, E).

### SIRT2 positively regulates the transcription factor Snail

**Fig. 7:**
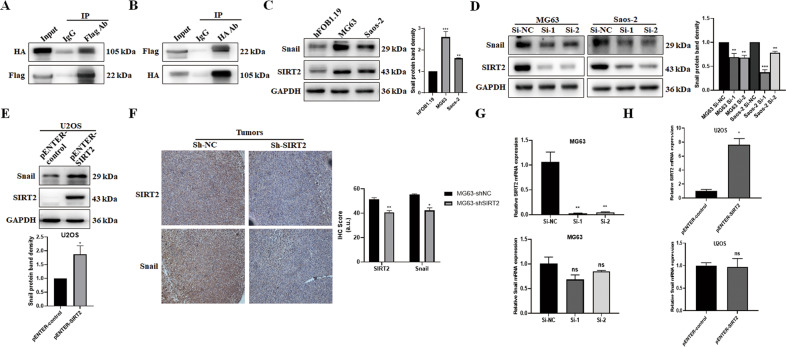
SIRT2 positively regulates the expression of Snail. **A**, **B** The pENTER-SIRT2-c-Flag-His and pcDNA3.1-Snail-c-HA plasmids were transfected into MG63 cells, and the binding of exogenous SIRT2 and Snail was detected by Co-IP. **C** Western blot was used to detect Snail expression in MG63 and Saos-2 cells. Data from a representative experiment of at least three independent samples are shown. **D** The expression of Snail was detected after SIRT2 knockdown in MG63 and Saos-2 cells. Data from a representative experiment of at least three independent samples are shown. **E** The expression of Snail was detected after SIRT2 overexpression in U2OS cells. **F** Immunohistochemical staining of SIRT2 and Snail was performed on the tumor tissues of the MG63-shSIRT2 group and control group. Immunohistochemical scores are summarized in a histogram format. **G**, **H** qRT-PCR was employed to detect Snail mRNA levels with SIRT2 knockdown or overexpression. (*P < 0.05, **P < 0.01, ***P < 0.001).

To explore the mechanism by which SIRT2 promotes the invasion and metastasis of osteosarcoma cells, we used the BioGRID database (https://thebiogrid.org/116593/table/homo-sapiens/sirt2.html) and HitPredict database (http://www.hitpredict.org/htp_int.php?Value=5142) to search for the molecules that interact with SIRT2. Both databases showed that SIRT2 may interact with the transcription factor Snail. We overexpressed SIRT2 (pENTER-SIRT2-c-Flag-His) and Snail (pcDNA3.1-Snail-c-HA) in MG63 cells and performed co-immunoprecipitation (Co-IP). We showed that SIRT2 bound to Snail (Fig. 7A, B). To clarify the regulatory relationship between SIRT2 and Snail, we detected the expression of Snail in osteosarcoma cells, and Snail expression was upregulated in MG63 and Saos-2 cells (with high SIRT2 expression) compared with hFOB1.19 control cells (with low SIRT2 expression) (Fig. 7C). In addition, the expression of Snail was decreased after SIRT2 knockdown in MG63 and Saos-2 cells (Fig. 7D). Overexpression of SIRT2 in U2OS cells upregulated the expression of Snail (Fig. 7E). We performed histochemical staining of SIRT2 and Snail in the tumor tissues of nude mice subcutaneously injected with MG63-shSIRT2 or control cells, and showed that the Snail staining was weakened after SIRT2 knockdown (Fig. 7F). To explore whether SIRT2 regulates Snail at the transcriptional level, qRT-PCR was employed. Knockdown or overexpression of SIRT2 did not affect the mRNA level of Snail (Fig. 7G, H). In summary, our data confirm the positive regulation of Snail by SIRT2 in vitro and in vivo.

### SIRT2 upregulates Snail by inhibiting its degradation

**Fig. 8:**
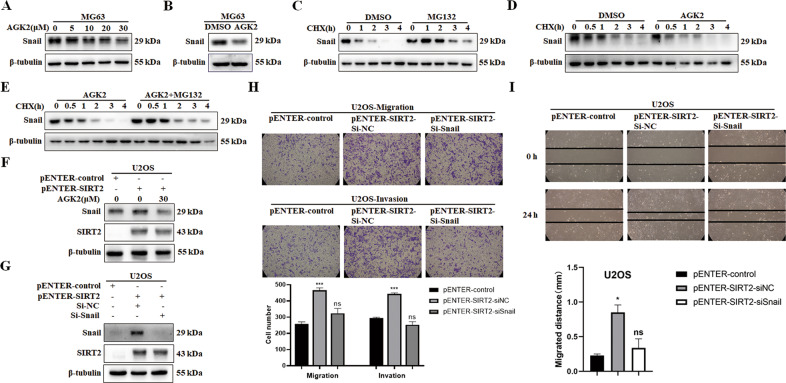
Inhibition of SIRT2 activity downregulates Snail expression through the proteasomal degradation. **A** Treatment of MG63 cells with AGK2 showed a dose-dependent decrease in Snail expression. Data from a representative experiment of at least three independent samples are shown. **B** MG63 cells were treated with 30 μM AGK2 to detect the expression of Snail protein. **C** CHX was used to inhibit the synthesis of protein, and MG132 was used to treat MG63 cells. Data from a representative experiment of at least three independent samples are shown. **D** CHX and AGK2 were used to treat MG63 cells. Data from a representative experiment of at least three independent samples are shown. **E** After the combined treatment with MG132 and AGK2, Snail expression was detected. Data from a representative experiment of at least three independent samples are shown. **F** SIRT2-overexpressing cells were treated with AGK2 and Snail levels were detected. Data from a representative experiment of at least three independent samples are shown. **G** SIRT2-overexpressing cells were transfected with Snail siRNA and Snail levels were detected. Data from a representative experiment of at least three independent samples are shown. **H**, **I** Transwell and wound-healing assay were used to analyze the effect of Snail knockdown on the migration and invasion of SIRT2-overexpressing cells. Results from three experiments are summarized in a histogram format. (*P < 0.05, ***P < 0.001).

We further explored whether SIRT2 regulated Snail at the protein level. AGK2 is a selective inhibitor of SIRT2 activity, and Snail expression was decreased when the cells were treated with AGK2 in a dose-dependent manner (Fig. 8A), suggesting that SIRT2 upregulates Snail through its deacetylase activity. We chose to treat cells with 30 μM AGK2 and further confirmed the inhibition of Snail (Fig. 8B). The protein synthesis inhibitor, cycloheximide (CHX) was used to treat MG63 cells to inhibit protein synthesis, and then the proteasome inhibitor, MG132 was used to treat the cells. The expression of Snail was obviously increased with MG132 treatment, indicating that Snail was degraded through the proteasome pathway (Fig. 8C). Compared with CHX treatment alone, the expression of Snail was decreased more quickly with AGK2 treatment, further indicating that SIRT2 upregulated Snail through its deacetylase activity (Fig. 8D). Combined treatment with MG132 and AGK2 rescued the decrease in Snail level caused by AGK2 treatment, confirming that SIRT2 upregulated Snail by inhibiting Snail’s proteasomal degradation (Fig. 8E). AGK2 treatment decreased the protein expression of Snail in SIRT2-overexpressing cells, which further suggests that SIRT2 regulates Snail via its deacetylase activity (Fig. 8F). We then knocked down Snail in SIRT2-overexpressing cells (Fig. 8G) and showed that Snail knockdown abrogated the promoting effect of SIRT2 on the migration and invasion of osteosarcoma cells (Fig. 8H, I), suggesting that SIRT2 promotes the invasion and metastasis of osteosarcoma cells via Snail.

## Discussion

Osteosarcoma is one of the leading causes of cancer-related death in adolescents [3, 21]. Osteosarcoma is highly aggressive and rapidly invades surrounding tissues and causes the development of early hematogenous metastases, with the most common site being the lungs [22]. Surgical resection combined with chemotherapy is effective in approximately 70% of patients with osteosarcoma, but the 5-year survival rate for osteosarcoma patients with metastases at diagnosis is only approximately 20% [23]. Osteosarcoma cells are prone to invasion and metastasis, which is the main reason for poor prognosis [5]. SIRT2 may act as an inducer or inhibitor in cancer, which may be related to cancer subtypes, subcellular localization, changes in deacetylase activity, and differences in substrate expression levels [17]. In this study, we aimed to explore the role of SIRT2 in the development and migration of osteosarcoma, which to date has remained unclear. We showed that the expression of SIRT2 but not SIRT1 was upregulated in osteosarcoma cells, which suggests that SIRT2 may contribute to the development of osteosarcoma. In addition, knockdown of SIRT2 inhibited osteosarcoma cell viability, migration and invasion, while overexpression of SIRT2 promoted osteosarcoma cell viability, migration and invasion.

During EMT of tumors, N-cadherin is upregulated and E-cadherin is downregulated. This “cadherin switch” reduces cell adhesion, enhances migration and invasion, and leads to a low survival rate [24, 25].The ECM is a complex network composed of extracellular macromolecules such as collagen and glycoproteins. The migration of normal cells is restricted by the ECM, while tumor cells and macrophages secrete matrix metalloproteinases. MMP2 and MMP9 degrade ECM to promote cancer cell invasion [26]. We showed that in osteosarcoma cells the expression of the mesenchymal markers N-cadherin and Vimentin and of MMP2 and MMP9 was all downregulated with SIRT2 knockdown, while the epithelial marker E-cadherin was decreased and N-cadherin, Vimentin, MMP2 and MMP9 were increased with SIRT2 overexpression. In addition, the enzymatic activity of MMP2 was increased with SIRT2 overexpression and decreased with SIRT2 knockdown. These results suggest that osteosarcoma cells activate EMT progression by upregulating SIRT2 to promote the “cadherin switch” and promote tumor invasion and metastasis.

To explore the mechanism by which SIRT2 regulates EMT, we searched the BioGRID database and HitPredict database for the molecules that interact with SIRT2. Snail was found to be one of the candidates. We proved the binding of SIRT2 and Snail in osteosarcoma cells and showed that SIRT2 positively regulated the expression of Snail. Studies have shown that Snail overexpression in a variety of tumors is associated with tumor grade, lymph node metastasis and tumor recurrence, and leads to poor prognosis [27]. Snail inhibits E-cadherin transcription by binding to the E-box of the E-cadherin promoter [28]. Snail also inhibits other epithelial molecules such as the tight junction proteins claudin, occludin and zona occludin-1 (ZO-1) [29]. Snail upregulates the expression of genes associated with aggressive phenotypes, such as N-cadherin, Vimentin, MMP2, and MMP9 [30–32]. Thus, we infer that SIRT2 promoted EMT by upregulating Snail.

The Snail protein is very unstable, with a half-life of approximately 25 minutes. Snail not only exists in the nucleus, but also enters the cytoplasm through nuclear export. Posttranslational modifications (PTMs), including phosphorylation and ubiquitination, affect Snail protein stability, subcellular localization and activity. Casein kinase 1 (CK1) first phosphorylates Snail at serine 107 (Ser107) and serine 104 (Ser104) [33]. Glycogen synthase kinase 3β (GSK-3β) then phosphorylates Snail Ser100 and Ser96 to enable it to exit the nucleus. Phosphorylation at Ser96 and Ser100 provides a recognition site for binding to the E3 ubiquitin ligase β-TrCP1, resulting in Snail ubiquitination and proteasomal degradation [34, 35]. We showed that SIRT2 inhibits Snail ubiquitination and degradation, ultimately stabilizes Snail. We suppose that SIRT2 may associate with CK1, GSK-3β or β-TrCP1 to inhibit the phosphorylation, nuclear export and ubiquitin-mediated degradation of Snail, or that SIRT2 directly deacetylates Snail to inhibit its degradation. Both of these possibilities need to be further explored.

In this study, SIRT2 showed increased levels in the osteosarcoma cell lines MG63 and Saos-2. Its overexpression enhanced and its knockdown restrained cell viability, migration, invasion and EMT. SIRT2 interacted with the transcription factor Snail and inhibited the proteasomal degradation of Snail to promote EMT. Snail knockdown reduced SIRT2-promoted cell invasion and metastasis. SIRT2 knockdown inhibited both tumorigenesis and lung and liver metastasis of osteosarcoma via Snail in vivo. Therefore, SIRT2 might be a promising therapeutic target for treating osteosarcoma.

## Methods and materials

### Cell culture

The human osteoblast cell line hFOB1.19 was purchased from the Cell Bank of the Typical Culture Preservation Committee of the Chinese Academy of Sciences. The human OS cell line Saos-2 was purchased from Shanghai Gaining Biotechnology. The human OS cell lines HOS, MG-63 and U2OS were purchased from the American Type Culture Collection (ATCC). The hFOB1.19 cells were maintained in DMEM and Ham’s F12 medium (DMEM/F12, Gibco BRL, USA) (1:1) with 0.3 mg/mL G418. HOS cells were cultured in Dulbecco’s modified Eagle medium (DMEM, Gibco BRL, USA). MG63 cells were cultured in RPMI 1640. Saos-2 and U2OS cells were cultured in McCoy’s 5 A medium. All cells were supplemented with 10% FBS (Gibco BRL, USA), penicillin (100 U/mL) and streptomycin (100 mg/mL). The hFOB1.19 cells were cultured in 5% CO_2_ at 33.5 °C, and the other cells were cultured in a humidified atmosphere of 5% CO_2_ at 37 °C.

### siRNA and plasmid transfection

Specific small interfering RNA (siRNA) targeting SIRT2, Snail and negative control siRNAs were purchased from GenePharma (China). MG63 and Saos-2 cells were seeded in 6-well plates, and the cells were transfected with 20 nM siRNAs using jetPRIME transfection reagent according to the manufacturer’s instructions. For overexpression experiments, U2OS cells were transfected with the pENTER-SIRT2-c-Flag-His plasmid using Lipofectamine 2000. The siRNA sequences were as follows:

si-SIRT2-1: 5'-CCTAGAGGCCAAGGCTTAAdTdT-3';

si-SIRT2-2 (si-SIRT2): 5'-GAGGCCAUCUUUGAGAUCAGCUAUU-3';

si-Snail: 5'-CCUGUCAGAUGAGGACAGUGGGAAA-3';

si-NC: 5'-UUCUCCGAACGUGUCACGUTT -3'.

### Lentivirus infection

Lentivirus shRNAs targeting SIRT2 (shSIRT2) were purchased from GeneCopoeia (China). To acquire an MG63 cell line with stable SIRT2 knockdown (MG63-shSIRT2), MG63 cells were infected with shSIRT2 lentivirus (Multiplicity of infection, MOI = 80) using 5 µg/mL polybrene transfection reagent (GenePharma, China) and cells were selected with 0.5 µg /mL puromycin.

### Quantitative Reverse-Transcription PCR

RNA was extracted using TRIzol reagent (Invitrogen, USA) and reverse-transcribed to cDNA by the HiScript Q RT SuperMix (Vazyme, China) according to the manufacturer’s instructions. ChamQ SYBR qPCR Master Mix (Vazyme, China) was used to amplify the products, which were monitored on a CFX96 Touch Real-Time PCR detection system (Bio-Rad, USA). The PCR primer sequences were as follows:

SIRT2-F: CTGCGGAACTTATTCTCCCAGAC;

SIRT2-R: CCACCAAACAGATGACTCTGCG;

Snail-F: TTCTCACTGCCATGGAATTCC;

Snail-R: GCAGAGGACACAGAACCAGAAA;

GAPDH-F: GCACCGTCAAGGCTGAGAAC;

GAPDH-R: GCCTTCTCCATGGTGGTGAA.

### Western blot

Total cellular proteins were extracted with radioimmunoprecipitation assay (RIPA, Beyotime Biotechnology, China) lysis buffer with phenylmethanesulfonyl fluoride (PMSF, Beyotime Biotechnology, China) and protease inhibitor (BestBio, China) at a 100:1:1 ratio. Proteins were separated by polyacrylamide gel electrophoresis (PAGE) and transferred to PVDF membranes (Millipore, USA). The membrane was incubated with primary antibodies against: SIRT1 (1:1000, 13161-1-AP, Proteintech), SIRT2 (1:1000, 19655-1-AP, Proteintech), E-cadherin (1:1000, 20874-1-AP, Proteintech), EMT Antibody Sample Kit (1:1000, 9782, Cell Signaling), MMP2 (1:1000, 40994, Cell Signaling), MMP9 (1:1000,13667, Cell Signaling), Snail (1:1000, 3879, Cell Signaling), Snail (1:1000, 13099-1-AP, Proteintech), GAPDH (1:1000, 10494-1-AP, Proteintech), β-tubulin (1:1000, 86298, Cell Signaling), Flag (1:1000, 20543-1-AP, Proteintech) and HA (1:1000, 66006-2-Ig, Proteintech). The following day, the membranes were incubated with goat anti-mouse HRP-conjugated secondary antibody (ZB-2305) or goat anti-rabbit HRP-conjugated secondary antibody (ZB-2301) for 1 h. The proteins were visualized with the Immobilon Western Chemiluminescent HRP Substrate kit (Millipore, USA).

### Cell viability assay

Cell viability was assessed using a Cell Counting Kit 8 (CCK-8, BestBio, China) according to the manufacturer’s protocol. MG63 and Saos-2 cells with SIRT2 knockdown and U2OS cells with SIRT2 overexpression were seeded into a 96-well plate. At 0, 24, 48, 72 and 96 h after seeding (every 2 days of total 8 days for Saos-2 cells), 10 μL CCK-8 was added and incubated for 2 h at 37 °C, and then the absorbance of the solution was measured at a wavelength of 450 nm.

### Wound-healing assay

Cell migration was examined using the wound-healing assay. Briefly, the cells were plated and cultured overnight to approximately 80–90% confluence in a 6-well plate. A wound was created by scraping a straight scratch in the confluent cell layer with a pipette tip (200 μL). The cells were washed 3 times with PBS to remove the floating cells and serum-free culture medium was added. A computer-based microscopy imaging system was used to capture images of scratched positions at 0 h and 24 h. The migration distance was calculated and compared.

### Transwell migration and invasion assay

For the migration assay, 8 × 10^4^ MG63 or Saos-2 cells in 200 μL culture medium without FBS were seeded in Transwell chambers and the lower chambers were filled with 500 μL 20% FBS complete medium as a chemoattractant. After incubation for 36 h, cells that had migrated to the lower chamber were washed with PBS, fixed with 4% paraformaldehyde for 30 min and stained with 0.1% crystal violet for 10 min. The number of migrated cells was counted in five randomly selected fields under phase contrast microscope.

For the invasion assay, a layer of artificially reconstituted basement membrane material Matrigel was coated on the bottom of the upper surface of the cell membrane (the dilution ratio of Matrigel to serum-free medium was 1:6).

### Immunofluorescence

Transfected cells were seeded in 24-well plates containing slides. When the cells were 50% confluent, the cell slides were fixed in 4% paraformaldehyde for 15 min, and permeabilized with 0.2% Triton X-100 (Sigma, USA) for 30 min. The cell slides were blocked in 5% BSA for 1 h, and then incubated with N-cadherin (1:100, 22018-1-AP, Proteintech), Vimentin (1:100, 10366-1-AP, Proteintech) or E-cadherin (1:100, 20874-1-AP, Proteintech) antibody at 4 °C overnight. The following day, the cells were incubated with Dylight 488-conjugated goat anti-rabbit IgG (H + L) secondary antibody or Dylight 594-conjugated goat anti-mouse IgG (H + L) secondary antibody in the dark for 2 h. Next, DNA was stained with DAPI (Beyotime Biotechnology, China) for 5 min. Finally, the cell slides were sealed with antifade mounting medium and stored in the dark at 4 °C. Immunofluorescence images were obtained using a fluorescence microscope.

### Gelatin zymogram assay

When the cells grew to approximately 80% confluence, they were washed twice with sterile PBS and incubated in serum-free media at 37 °C in a CO_2_ incubator for at least 16-20 hours. The media was centrifuged (400×g for 5 min at 4 °C) to remove cells debris, and the supernatant was retained as a sample to be tested with a Gelatin Zymograpgy Analysis Kit (Real-Times Biotechnology, Beijing, China).

### Co-Immunoprecipitation

MG63 cells were co-transfected with SIRT2 (pENTER-SIRT2-c-Flag-His) and Snail (pcDNA3.1-Snail-c-HA) plasmids. Total proteins were extracted with weak RIPA lysis buffer (Beyotime, China). Protein A/G Plus Magnetic Beads (MedChemExpress, USA) were pre-incubated with the IgG (normal rabbit IgG, B900610, Proteintech), SIRT2 antibody or Snail antibody for 60 min on a spinning wheel at 4 °C. The bead-antibody complexes were washed three times and suspended with the protein lysate on a spinning wheel at 4 °C overnight. The beads were washed four times with PBST buffer, and were collected with a magnetic stand. The immunoprecipitates were then eluted by boiling with 2×loading buffer for Western blot analysis.

### Xenograft tumorigenesis and metastasis

Six-week-old male BALB/c athymic nude mice were subcutaneously injected into the flanks with 1 × 10^7^ MG63-shSIRT2 cells or MG63-shNC cells in 0.1 mL of PBS (*n* = 6 per group). Tumor growth was monitored, and tumor volume was measured every 2 days with calipers. The tumor volume was calculated with the formula: (length × width^2^)/2. Twelve days after injection, the mice were sacrificed, and the tumors were harvested and weighed. H&E staining, immunochemical staining of SIRT2 and Snail, and Western blot analysis of EMT-related proteins were performed. For the tumor metastatic assay, 2 × 10^6^/100 µL MG63-shSIRT2 cells or MG63-shNC cells were injected into the tail vein of 6-week-old male BALB/c athymic nude mice (*n* = 6 per group). Five weeks later, experimental lung and liver metastasis was determined. All animal experimental procedures and protocols were approved by the Experimental Animal Ethics Committee of Shandong University School of Medicine.

### Statistical analysis

Data are expressed as the mean ± standard deviation (SD). Statistical analysis was carried out using SPSS 20.0 (IBM, Chicago, IL, USA). The significant differences between the two groups were assessed by two-tailed Student’s *t-*test. Values of *P* < 0.05 were considered statistically significance.

## Supplementary information

checklist

Uncropped WB

## Data Availability

The experimental data sets generated and/or analyzed during the current study are available from the corresponding author upon reasonable request. No applicable resources were generated during the current study.
