# Supplementary material for: SIRT2 promotes the viability, invasion and metastasis of osteosarcoma cells by inhibiting the degradation of Snail
Source: Cell Death Dis. 2022 Nov 7;13(11):935. doi: 10.1038/s41419-022-05388-2 (PMC9640536; doi:10.1038/s41419-022-05388-2)
Supplement: Supplementary file 2 — Uncropped WB [file 41419_2022_5388_MOESM2_ESM.pptx]

## Slide 1
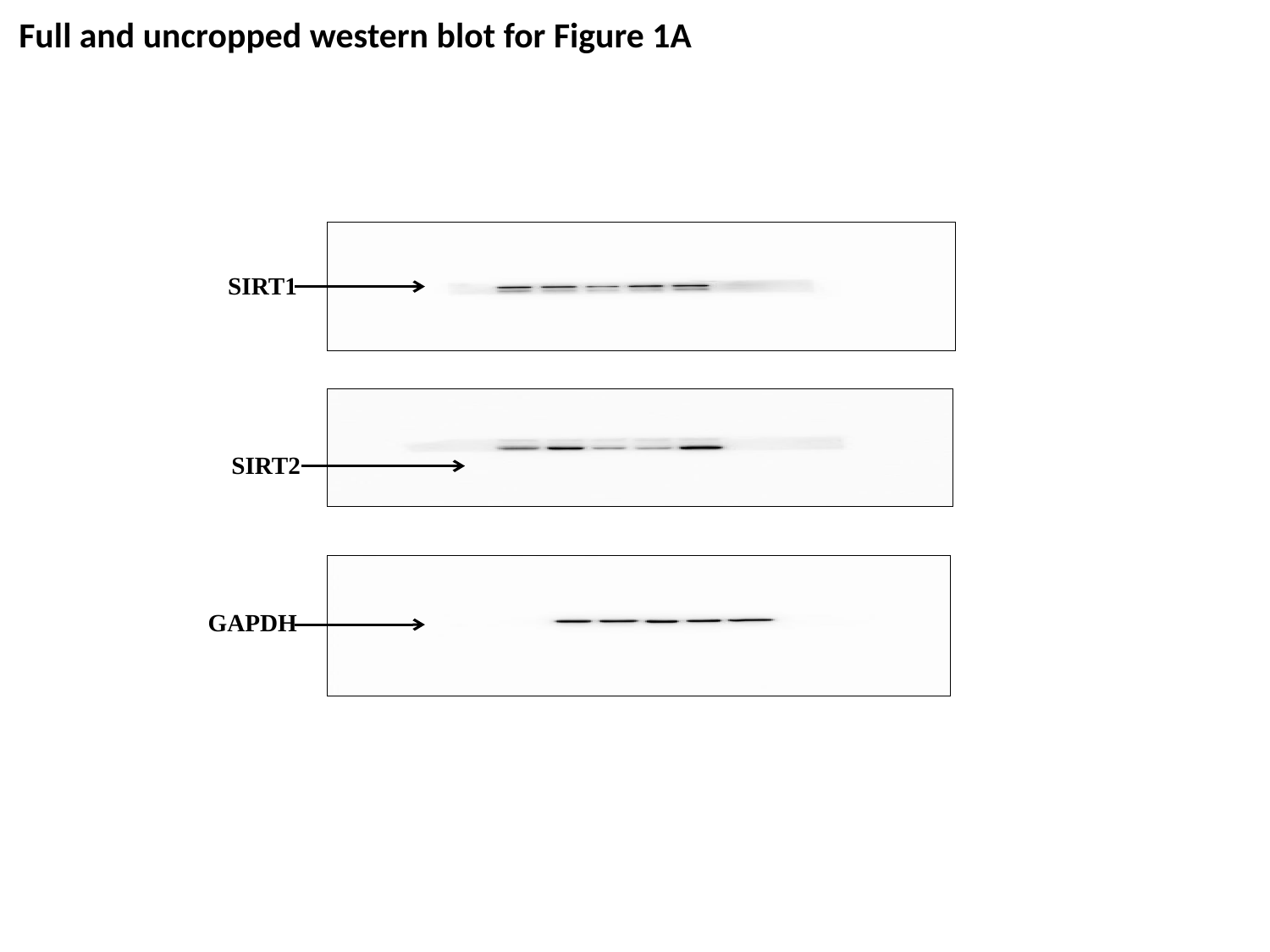

Full and uncropped western blot for Figure 1A
SIRT1
SIRT2
GAPDH

## Slide 2
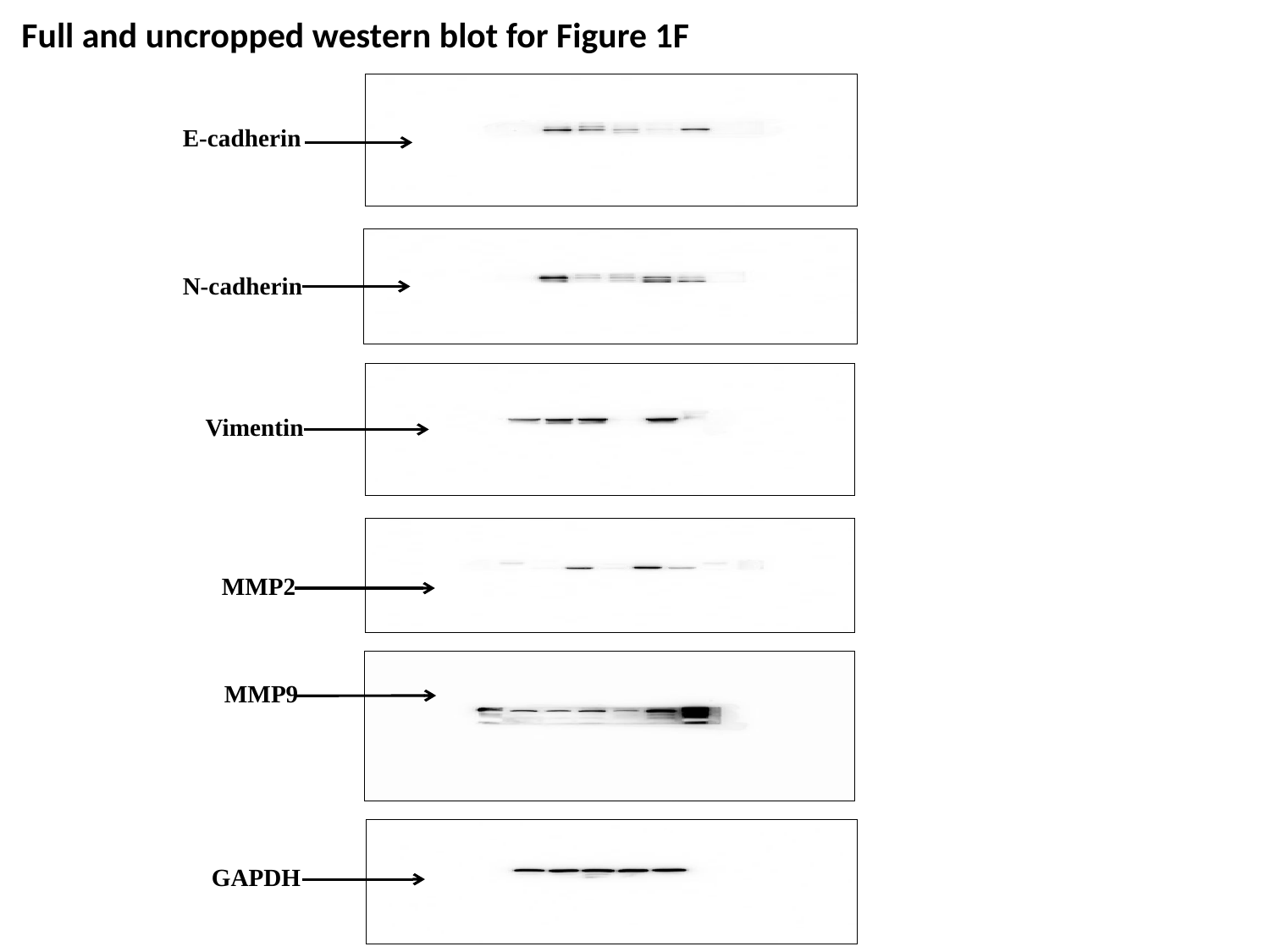

Full and uncropped western blot for Figure 1F
E-cadherin
N-cadherin
Vimentin
MMP2
MMP9
GAPDH

## Slide 3
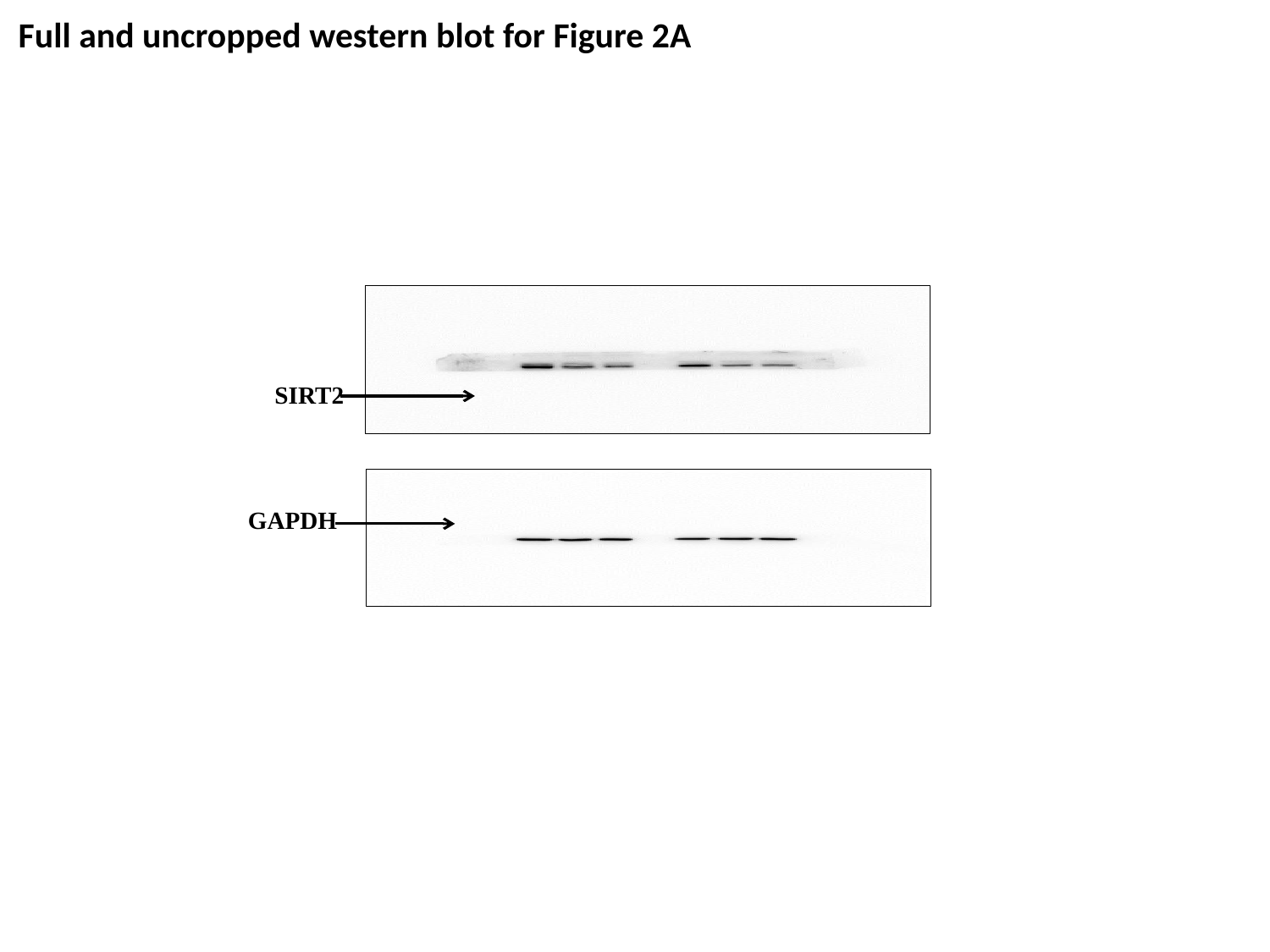

Full and uncropped western blot for Figure 2A
 SIRT2
GAPDH

## Slide 4
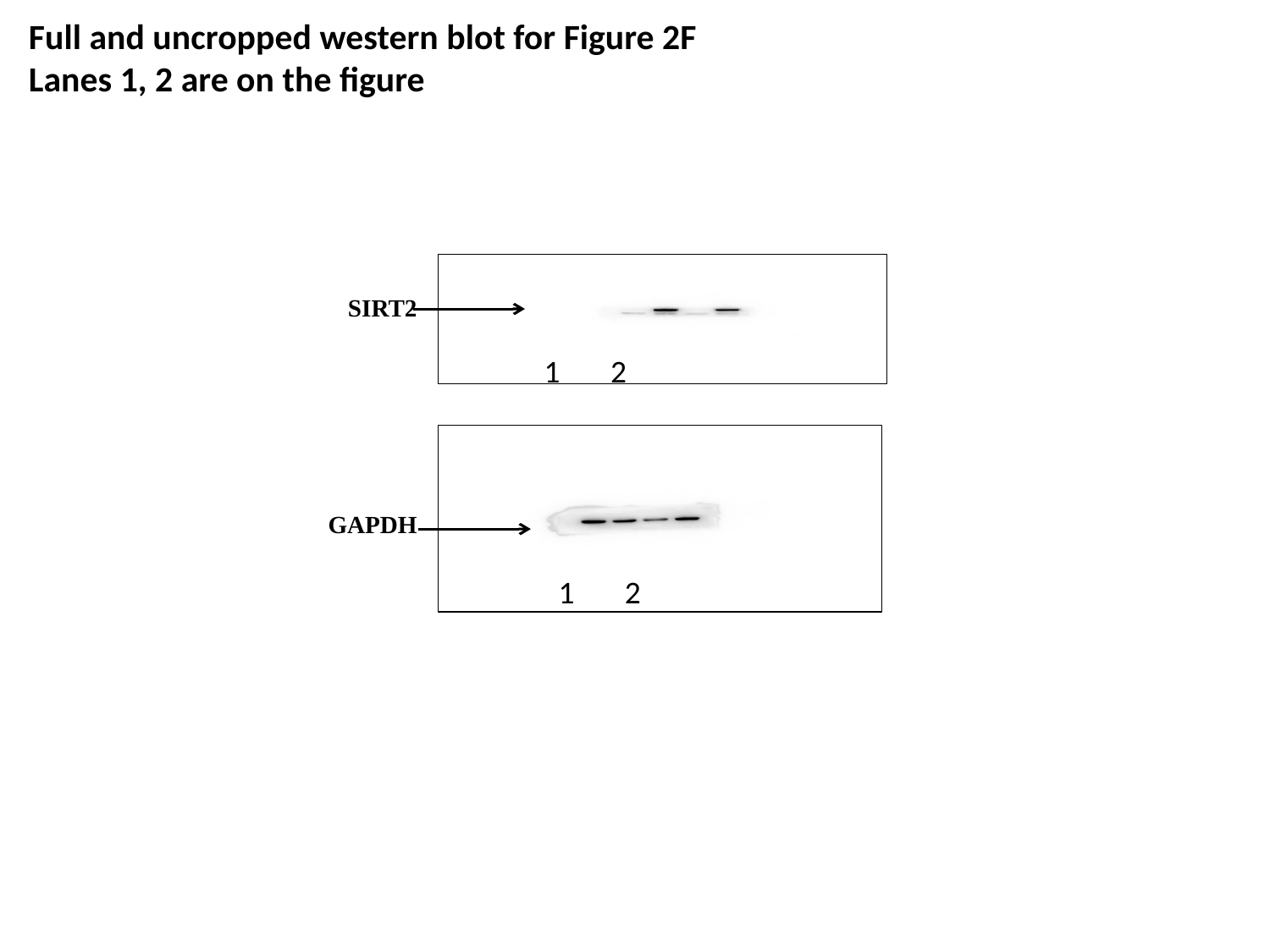

Full and uncropped western blot for Figure 2F
Lanes 1, 2 are on the figure
SIRT2
1 2
GAPDH
1 2

## Slide 5
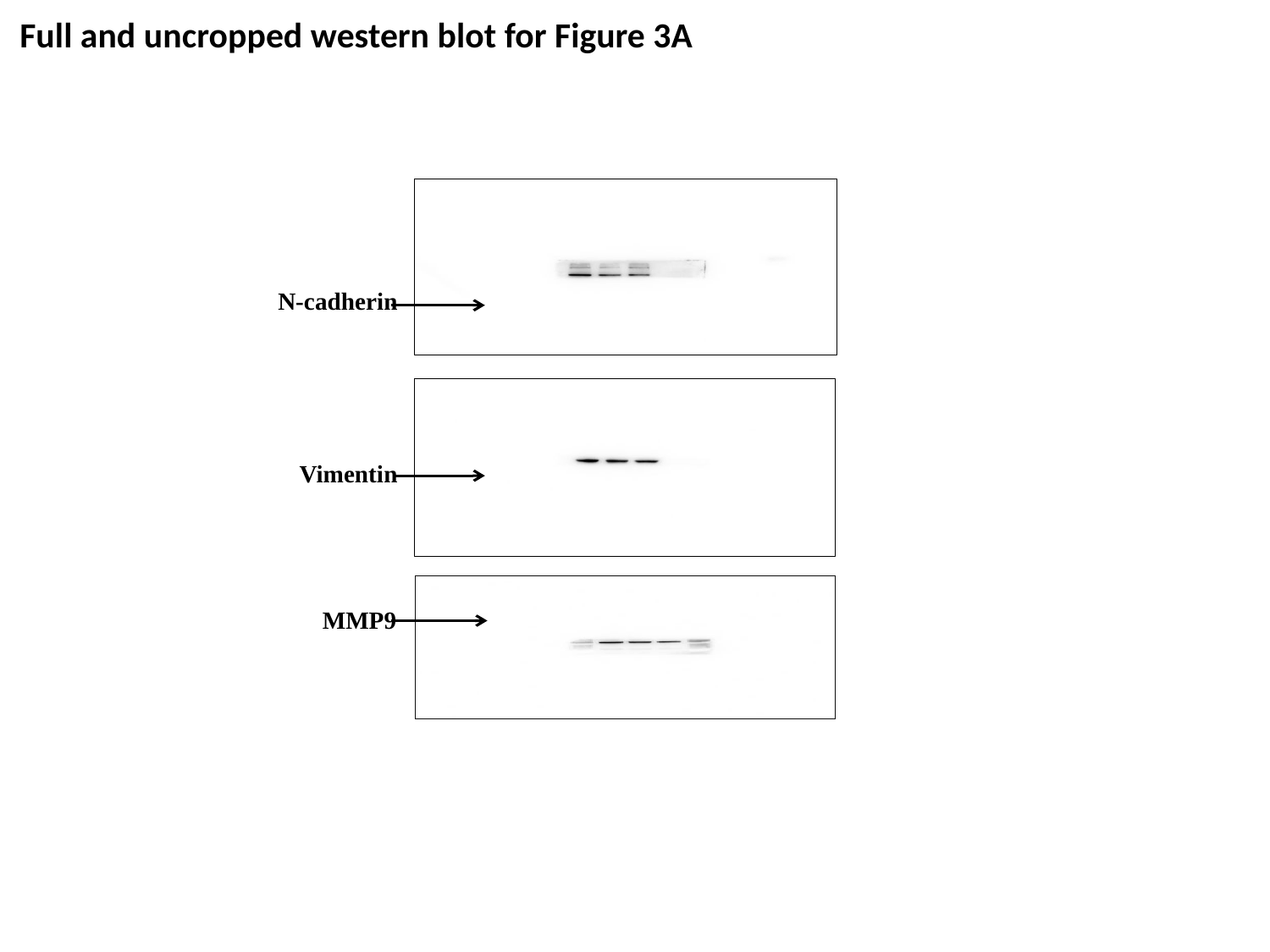

Full and uncropped western blot for Figure 3A
N-cadherin
Vimentin
MMP9

## Slide 6
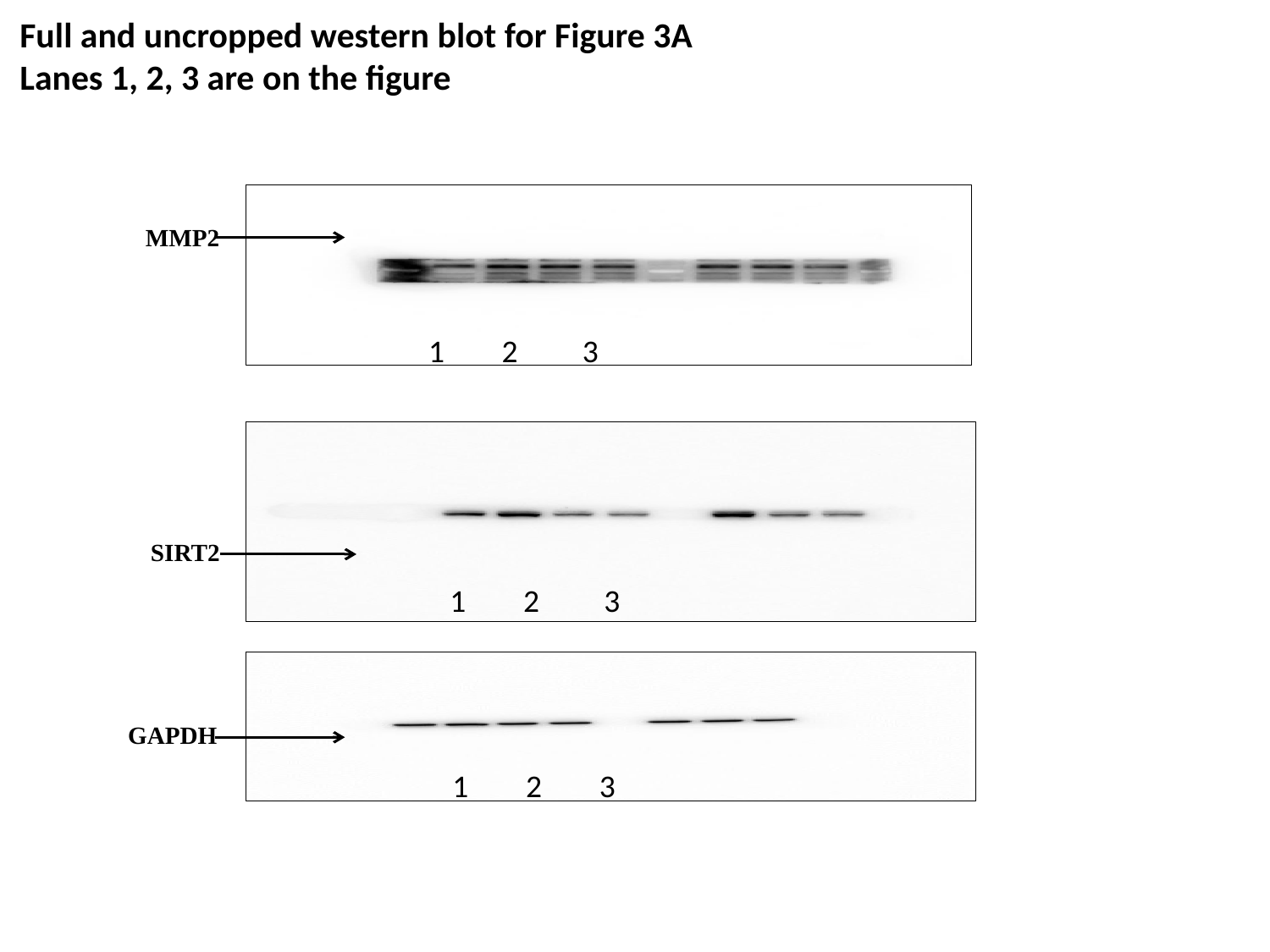

Full and uncropped western blot for Figure 3A
Lanes 1, 2, 3 are on the figure
MMP2
1 2 3
SIRT2
1 2 3
GAPDH
1 2 3

## Slide 7
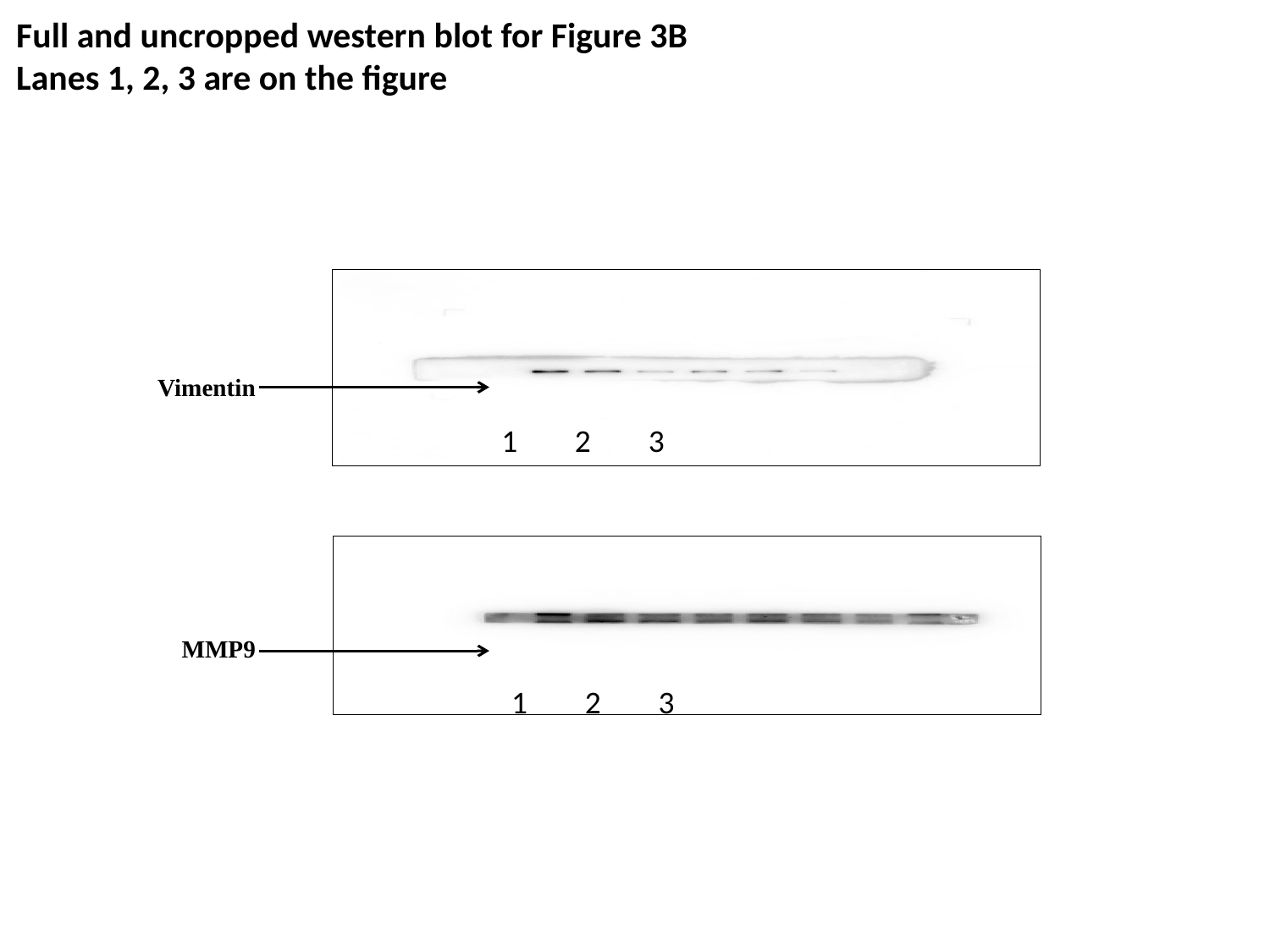

Full and uncropped western blot for Figure 3B
Lanes 1, 2, 3 are on the figure
Vimentin
1 2 3
MMP9
1 2 3

## Slide 8
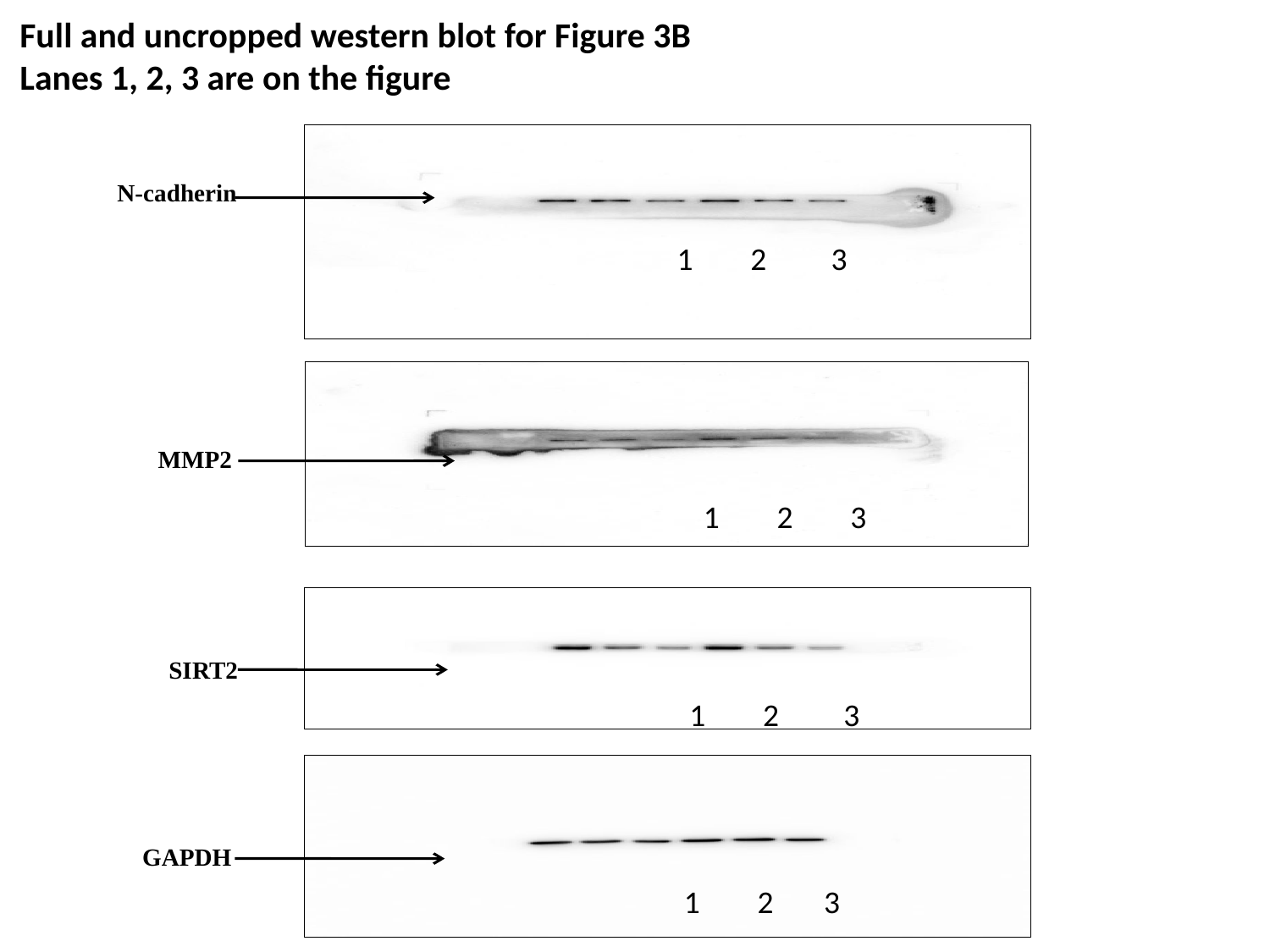

Full and uncropped western blot for Figure 3B
Lanes 1, 2, 3 are on the figure
N-cadherin
1 2 3
MMP2
1 2 3
SIRT2
1 2 3
GAPDH
1 2 3

## Slide 9
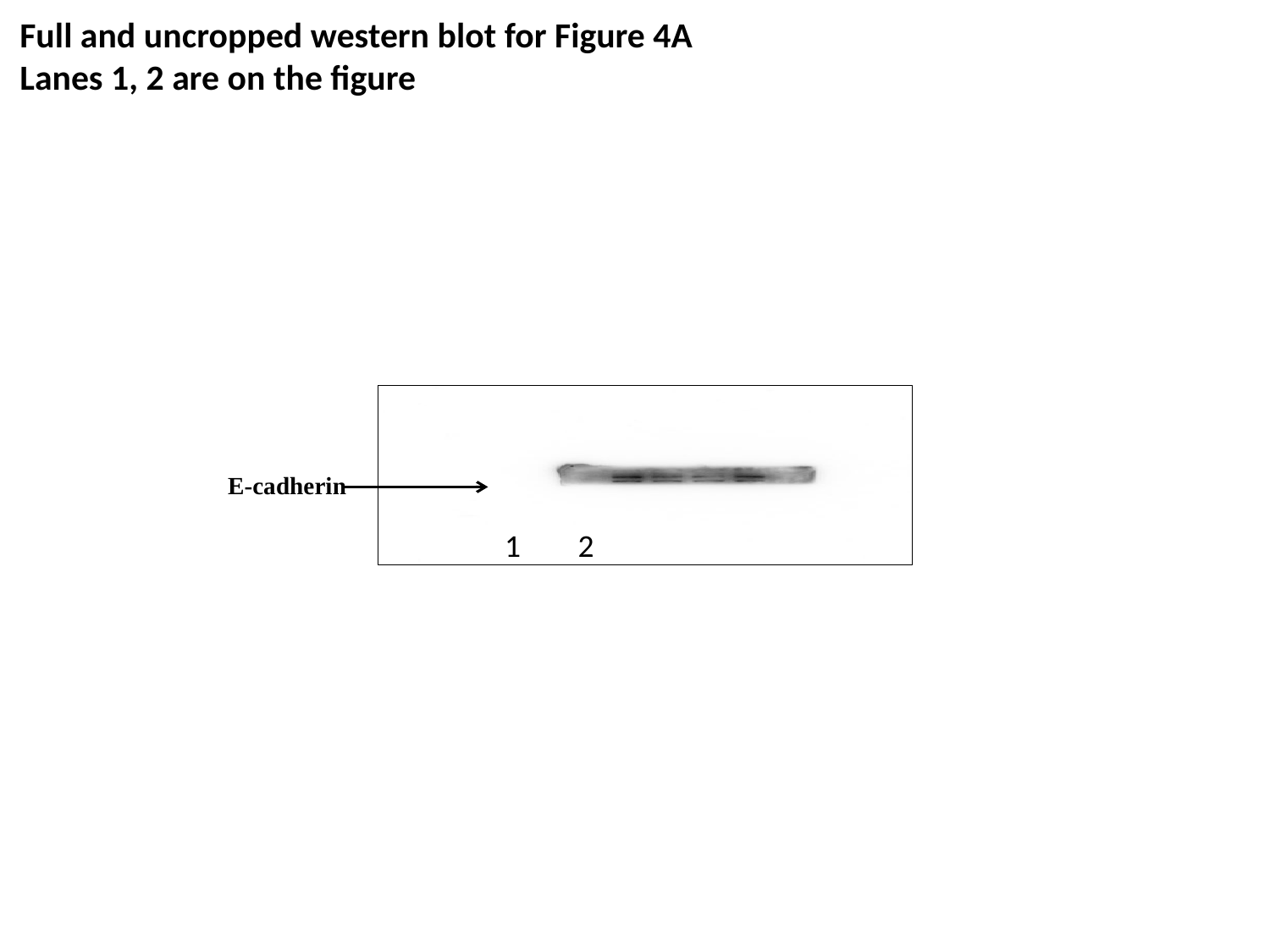

Full and uncropped western blot for Figure 4A
Lanes 1, 2 are on the figure
E-cadherin
1 2

## Slide 10
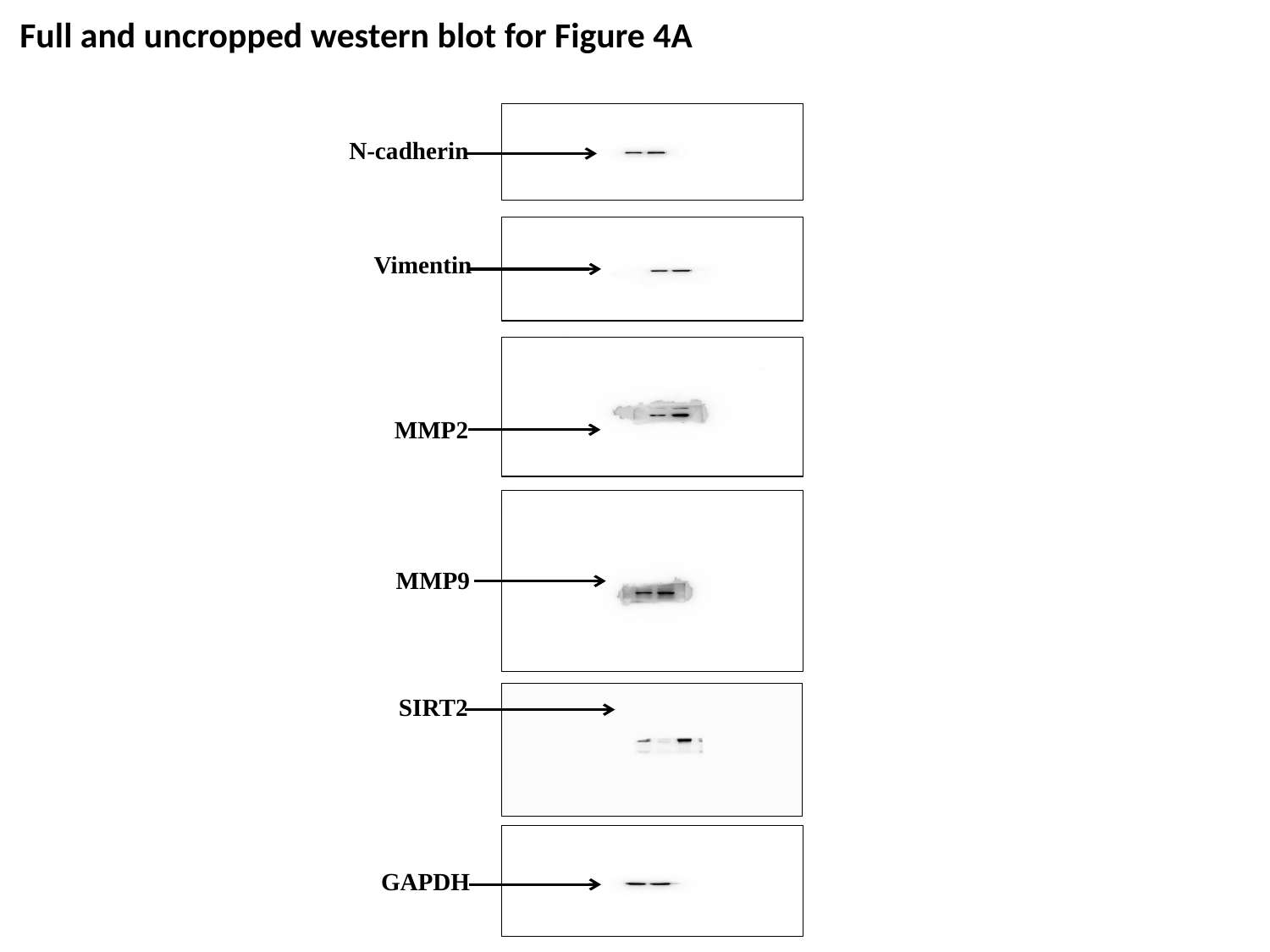

Full and uncropped western blot for Figure 4A
N-cadherin
Vimentin
MMP2
MMP9
SIRT2
GAPDH

## Slide 11
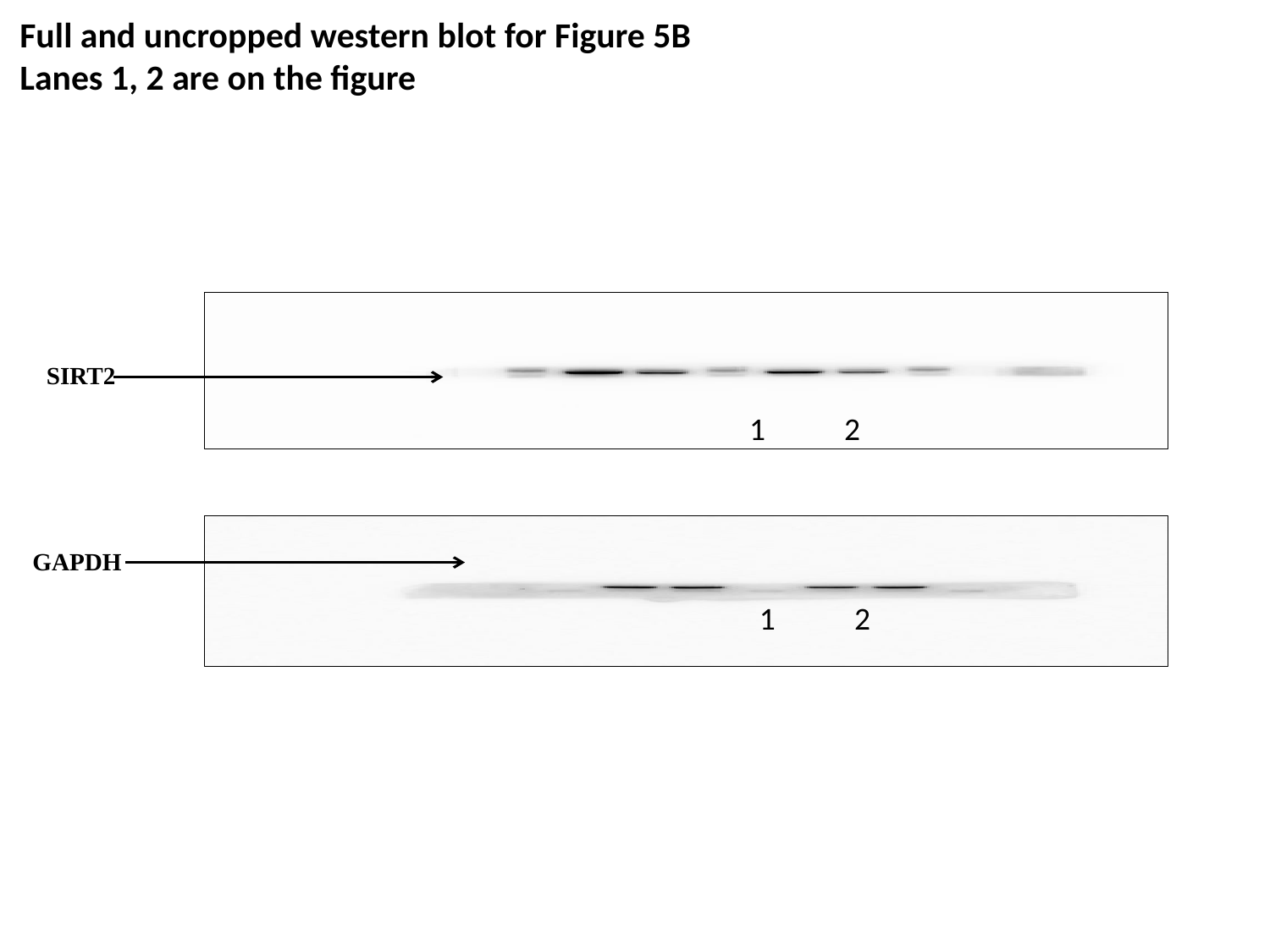

Full and uncropped western blot for Figure 5B
Lanes 1, 2 are on the figure
SIRT2
1 2
GAPDH
1 2

## Slide 12
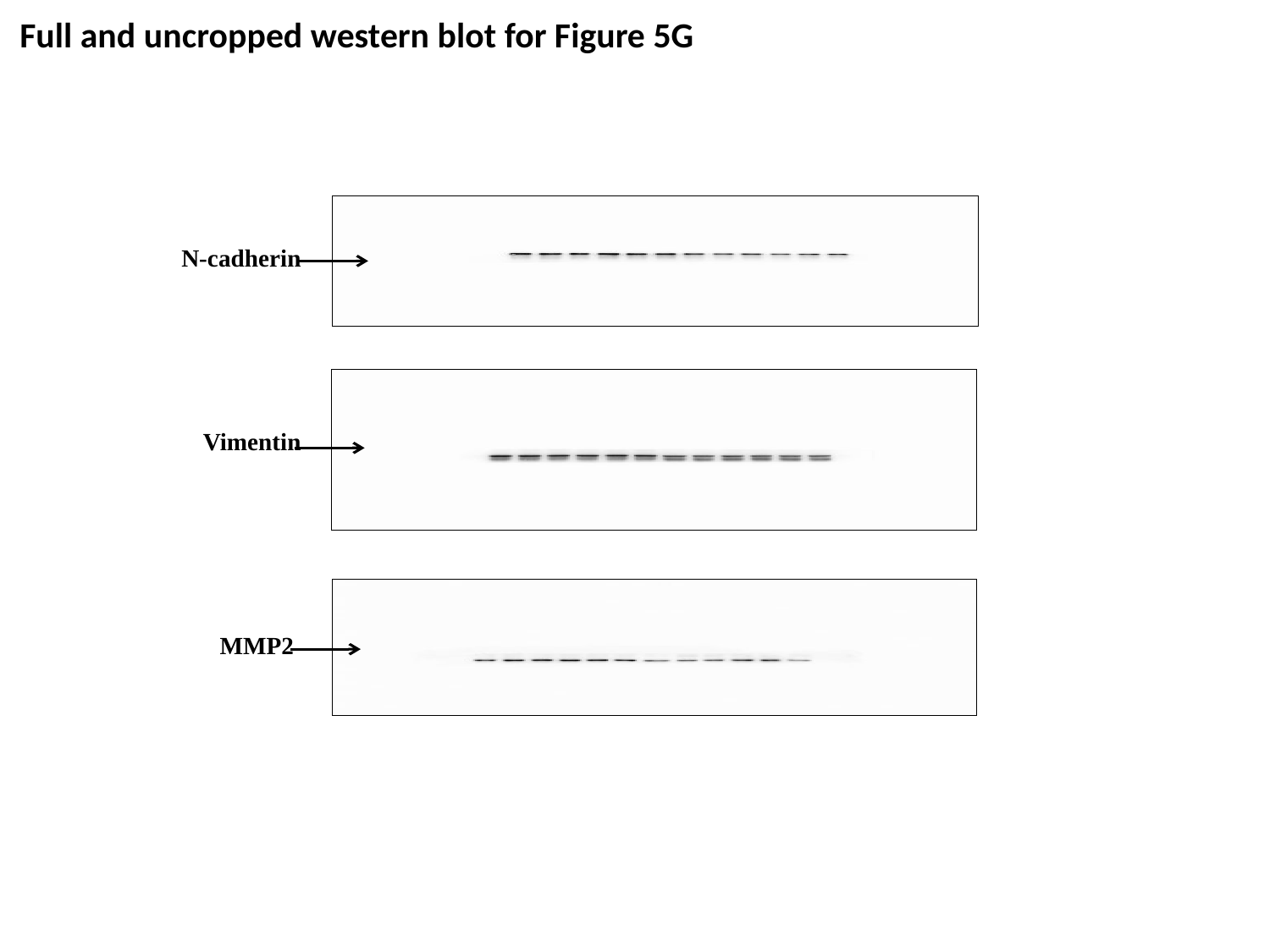

Full and uncropped western blot for Figure 5G
N-cadherin
Vimentin
MMP2

## Slide 13
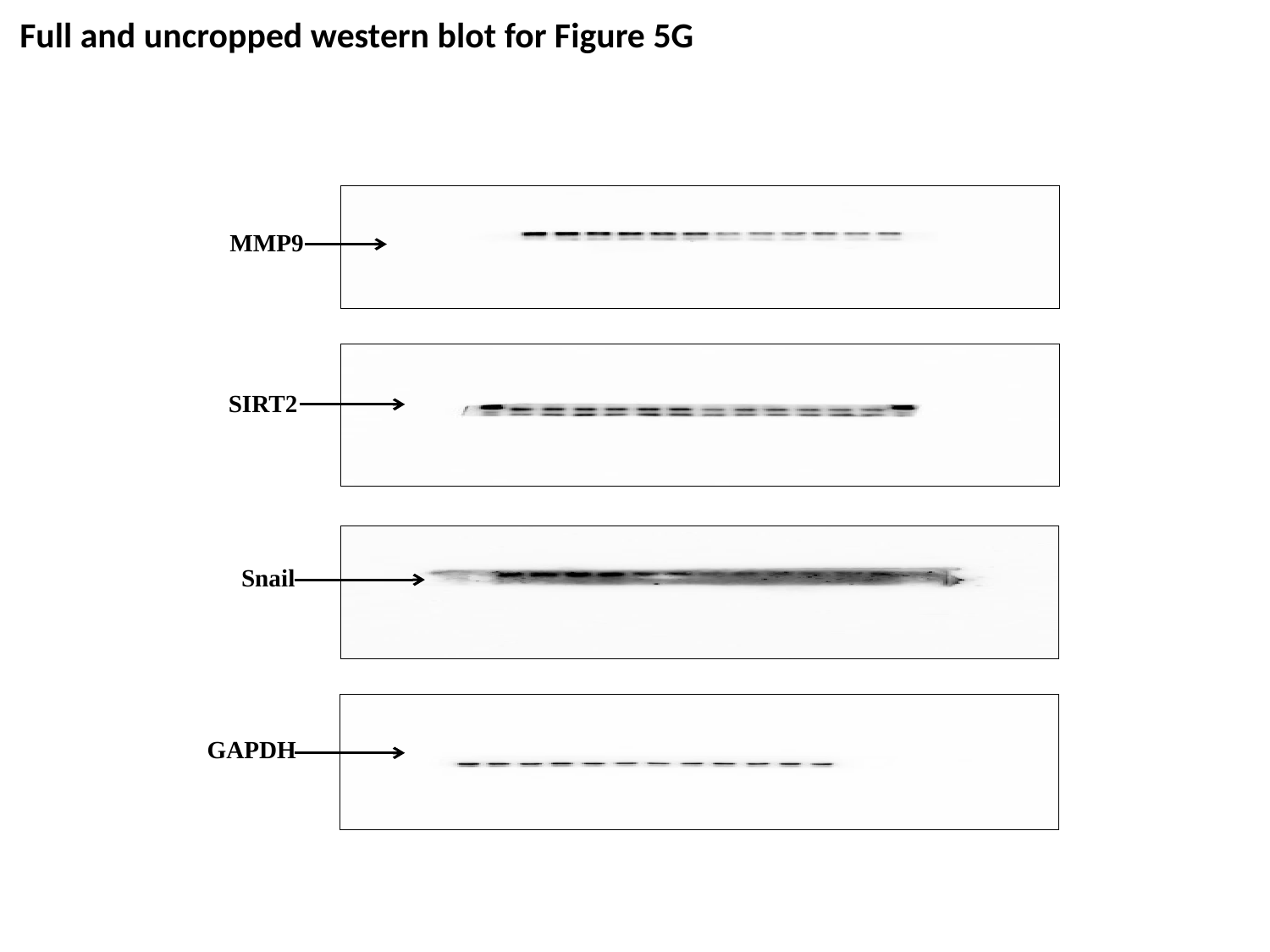

Full and uncropped western blot for Figure 5G
MMP9
SIRT2
Snail
GAPDH

## Slide 14
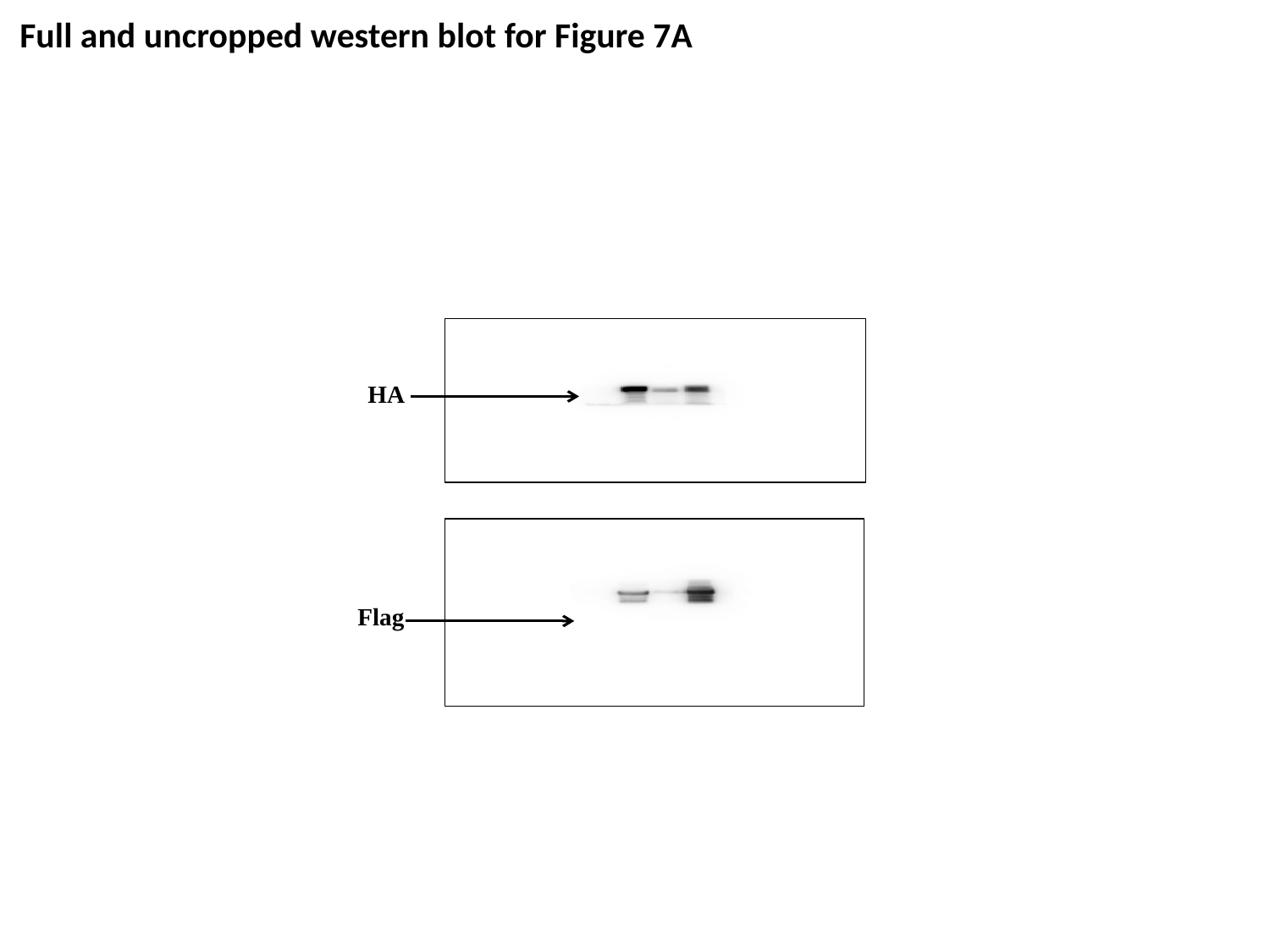

Full and uncropped western blot for Figure 7A
HA
Flag

## Slide 15
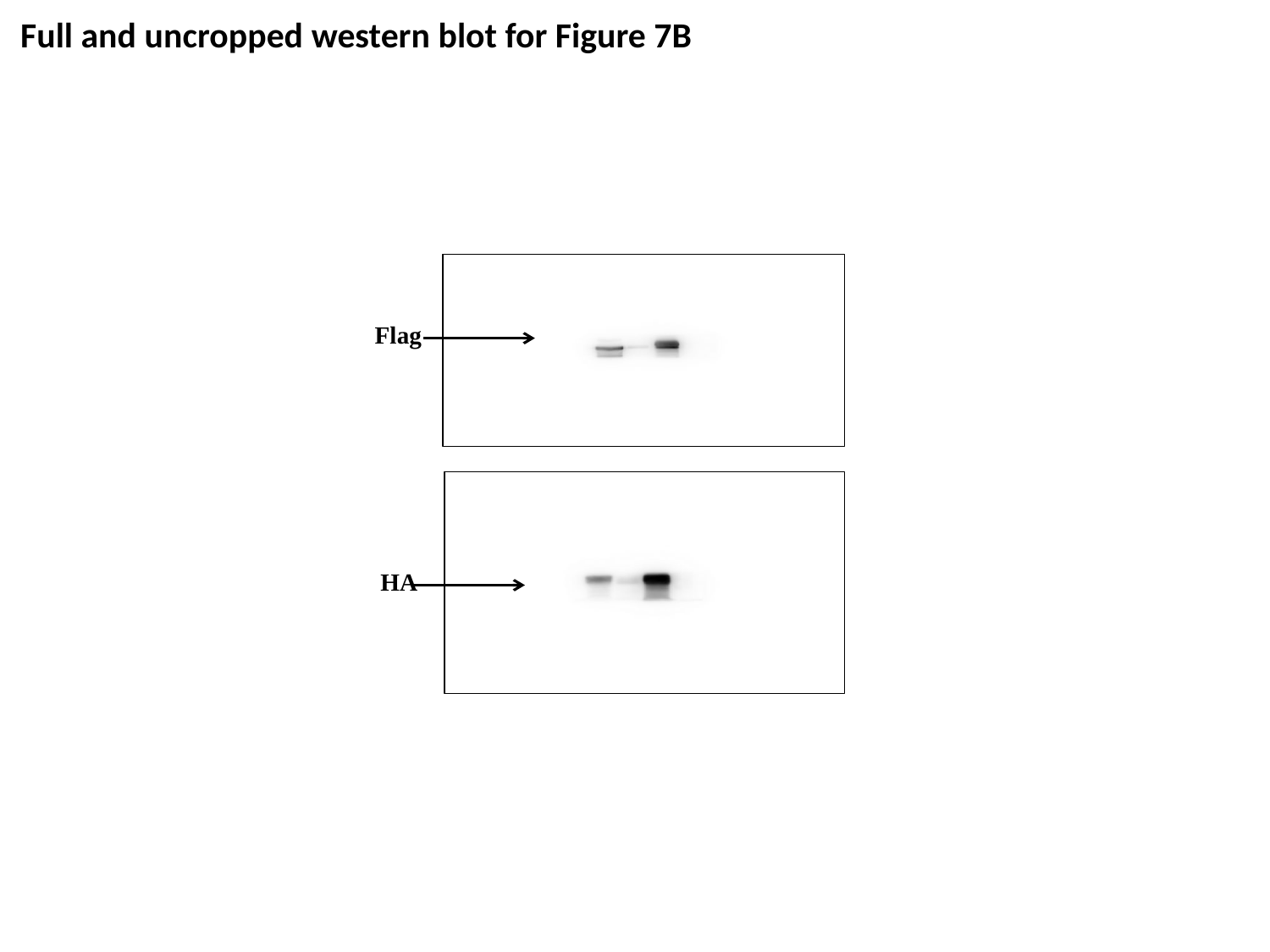

Full and uncropped western blot for Figure 7B
Flag
HA

## Slide 16
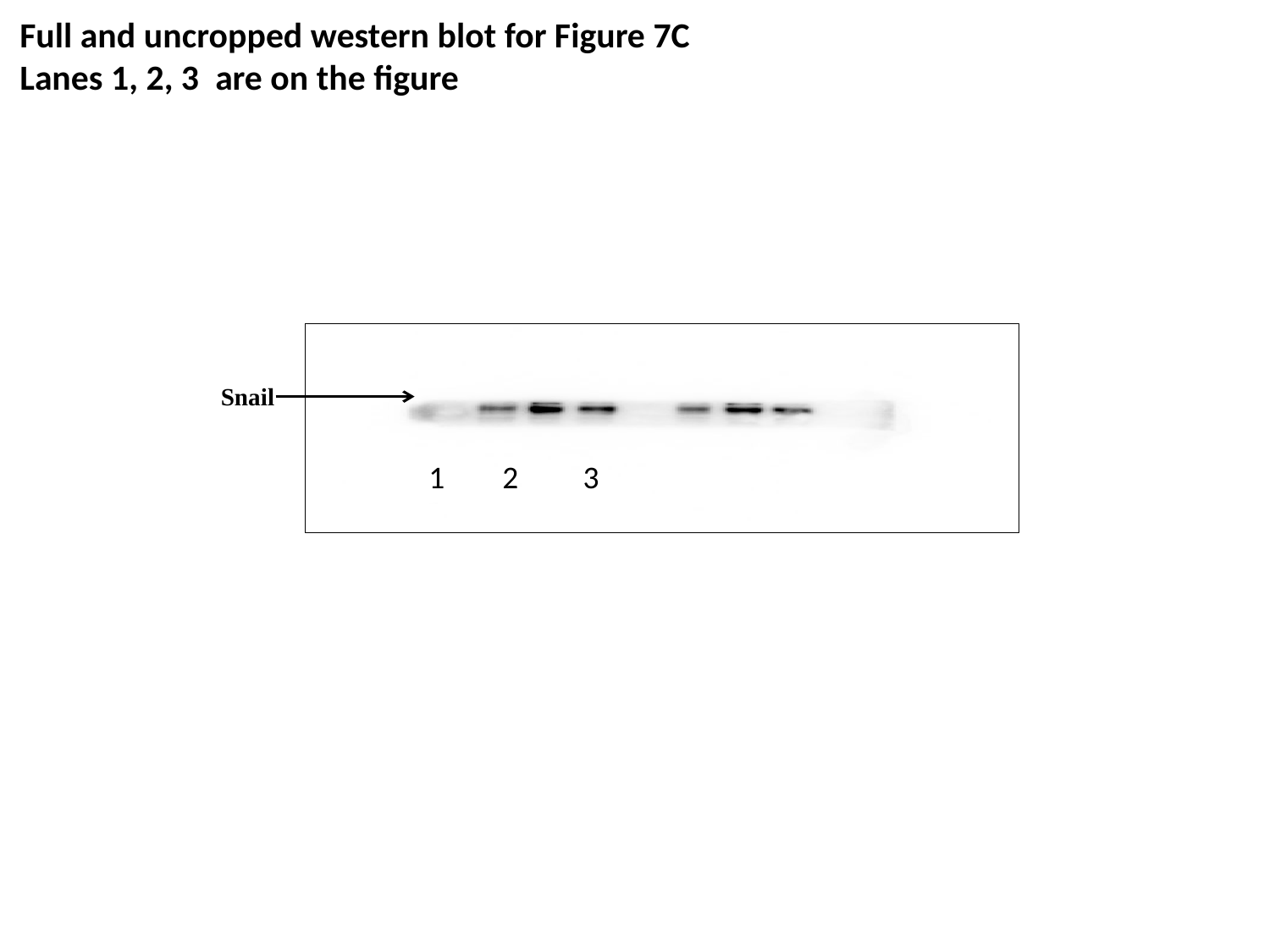

Full and uncropped western blot for Figure 7C
Lanes 1, 2, 3 are on the figure
Snail
1 2 3

## Slide 17
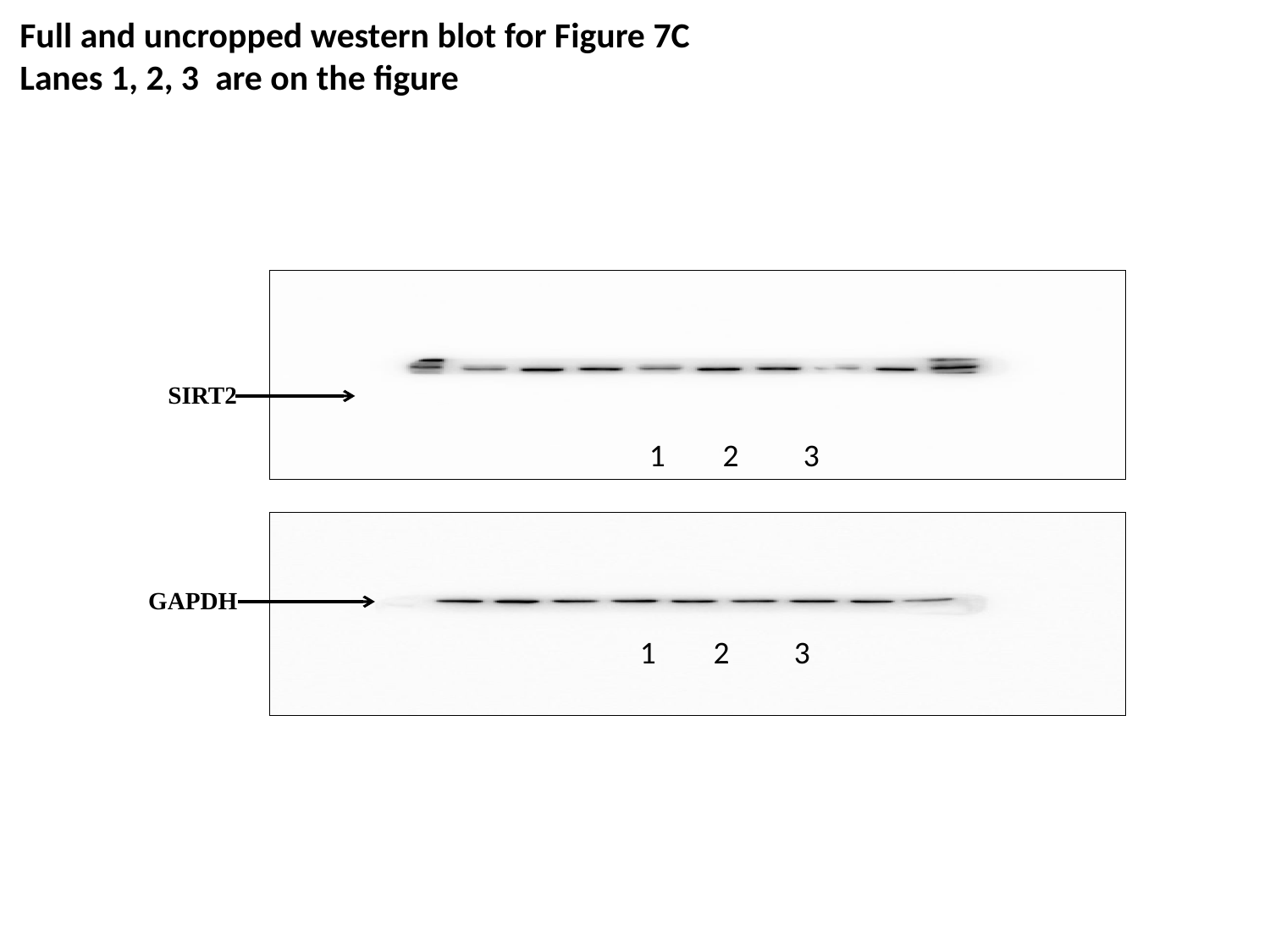

Full and uncropped western blot for Figure 7C
Lanes 1, 2, 3 are on the figure
SIRT2
1 2 3
GAPDH
1 2 3

## Slide 18
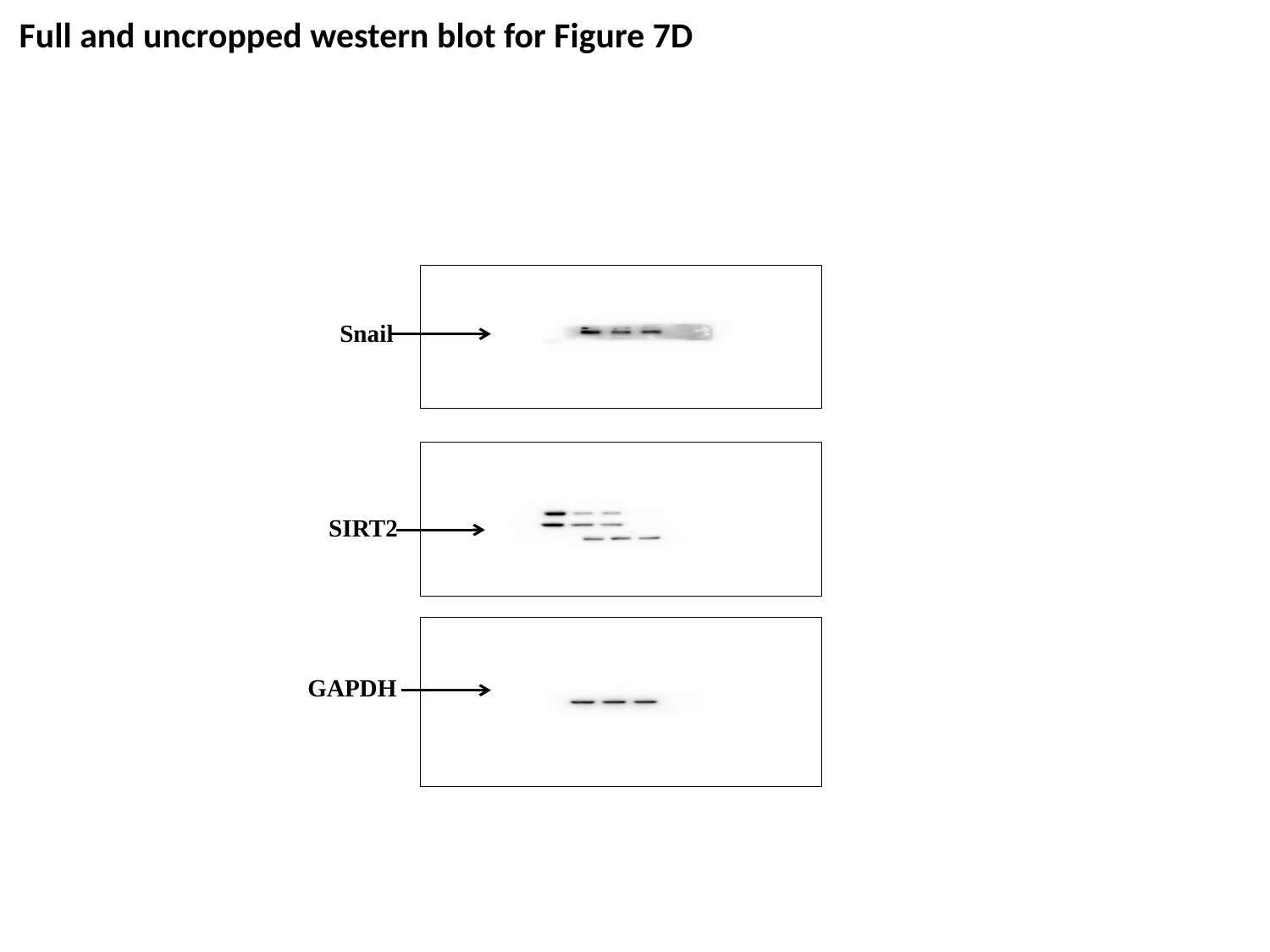

Full and uncropped western blot for Figure 7D
Snail
SIRT2
GAPDH

## Slide 19
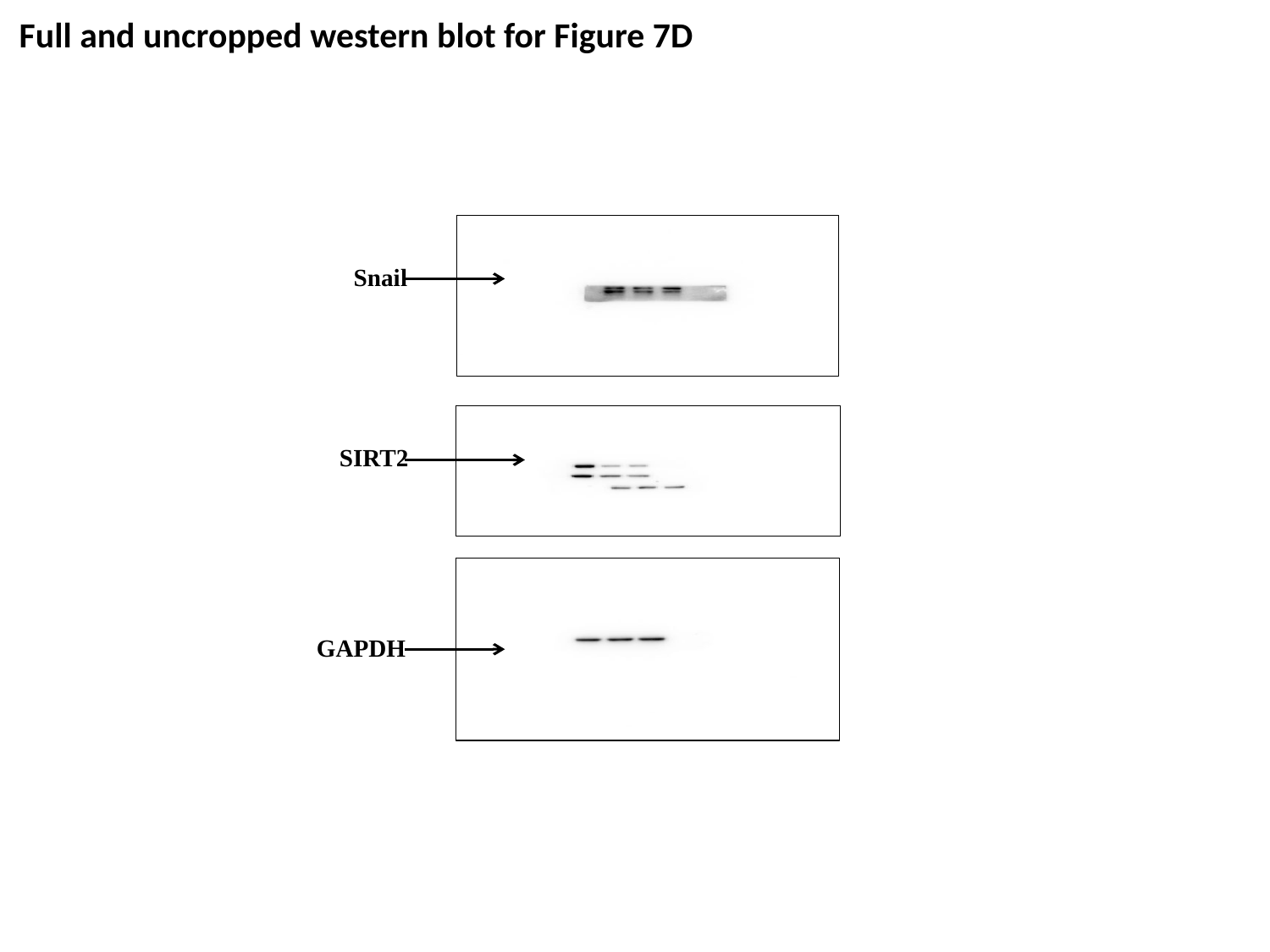

Full and uncropped western blot for Figure 7D
Snail
SIRT2
GAPDH

## Slide 20
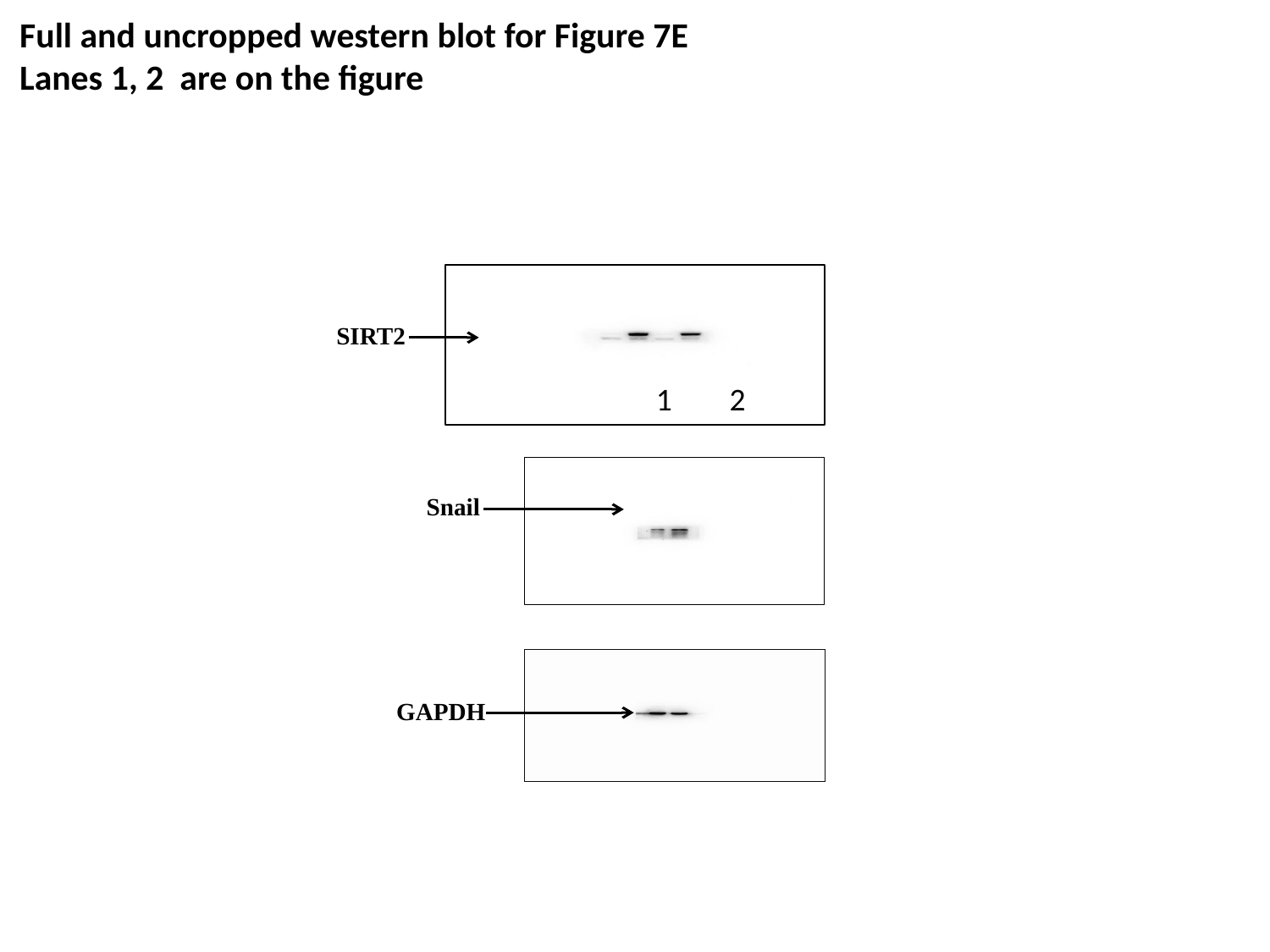

Full and uncropped western blot for Figure 7E
Lanes 1, 2 are on the figure
SIRT2
1 2
Snail
GAPDH

## Slide 21
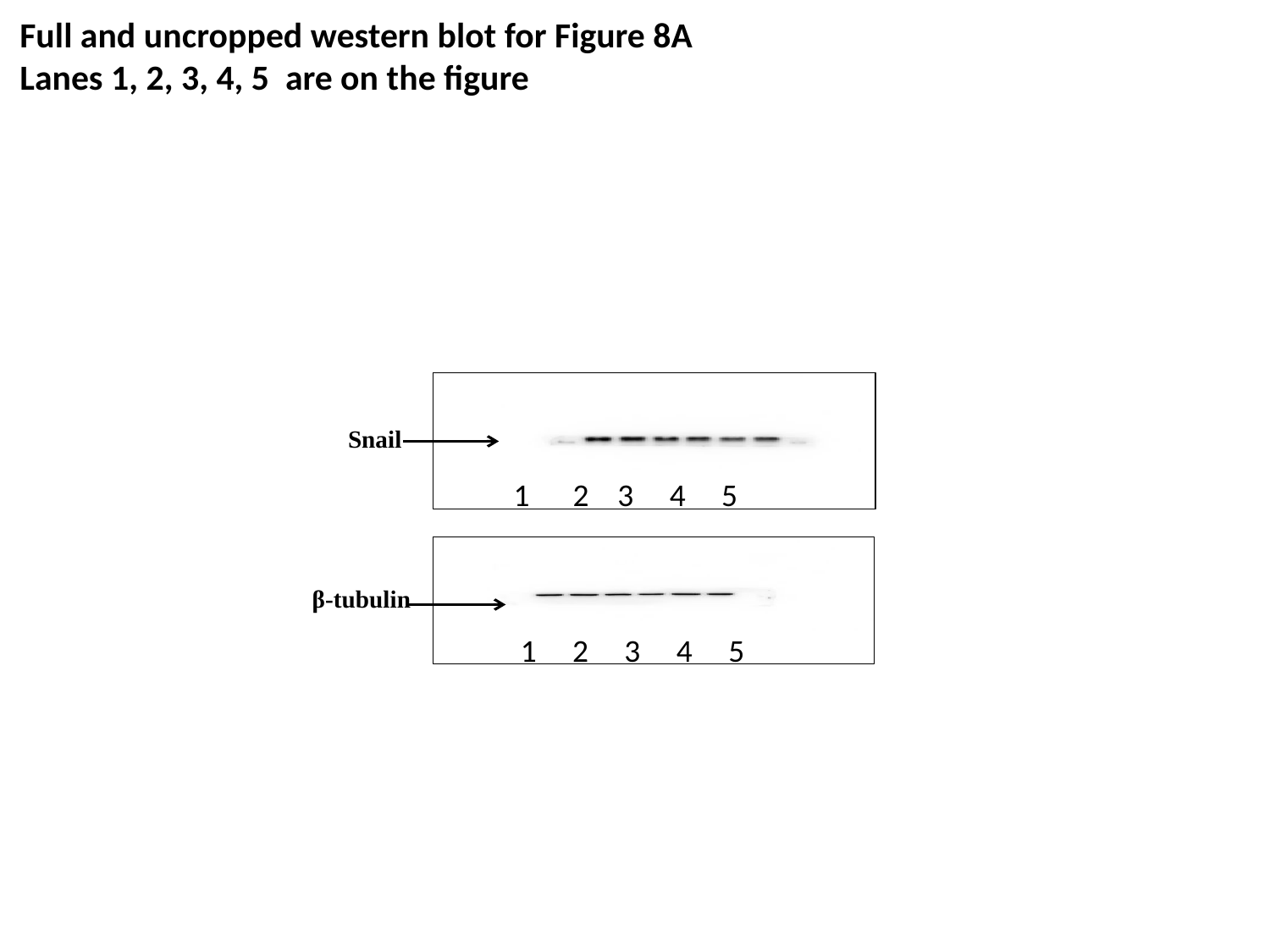

Full and uncropped western blot for Figure 8A
Lanes 1, 2, 3, 4, 5 are on the figure
Snail
Snail
1 2 3 4 5
β-tubulin
1 2 3 4 5

## Slide 22
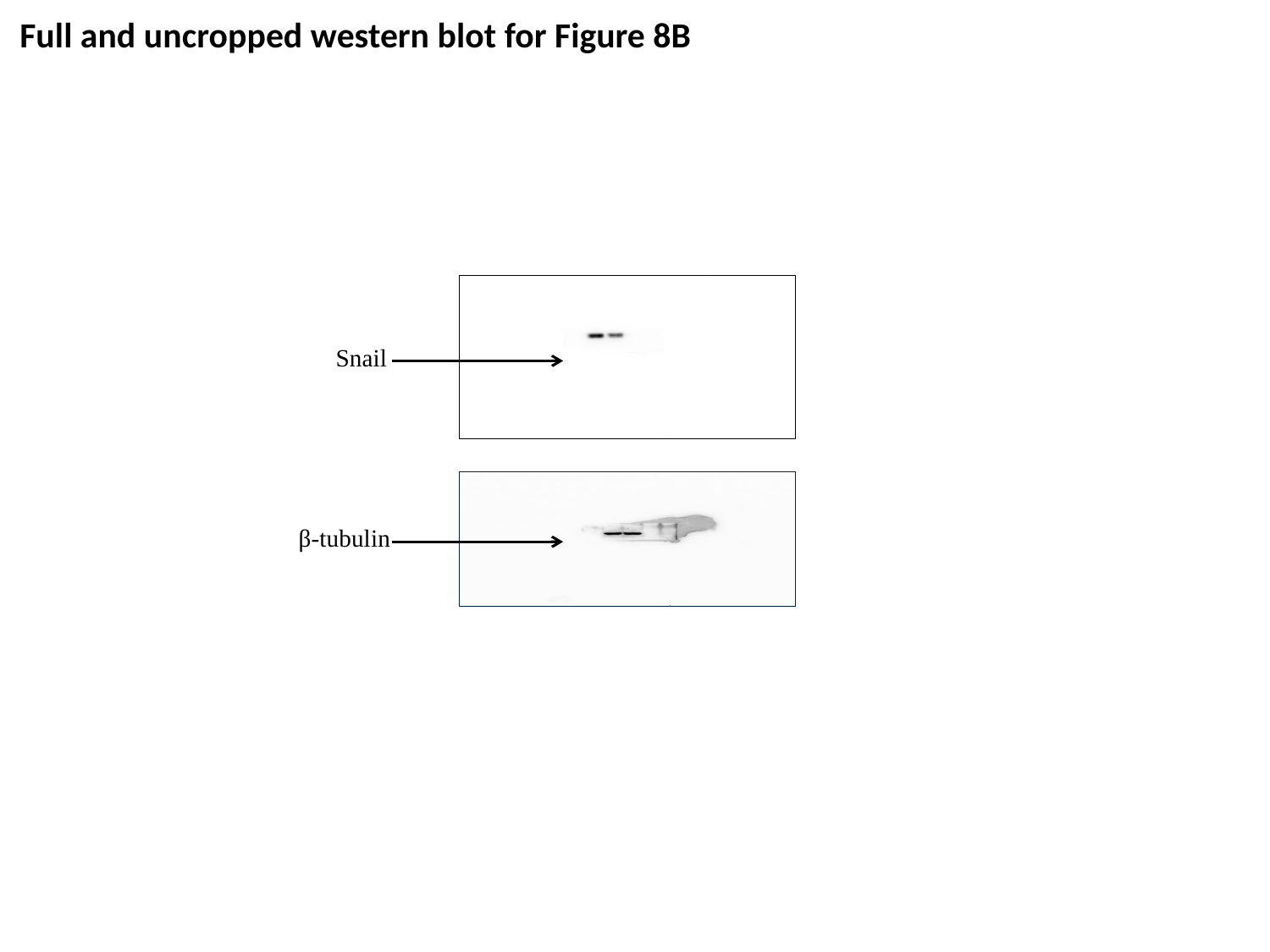

Full and uncropped western blot for Figure 8B
Snail
β-tubulin

## Slide 23
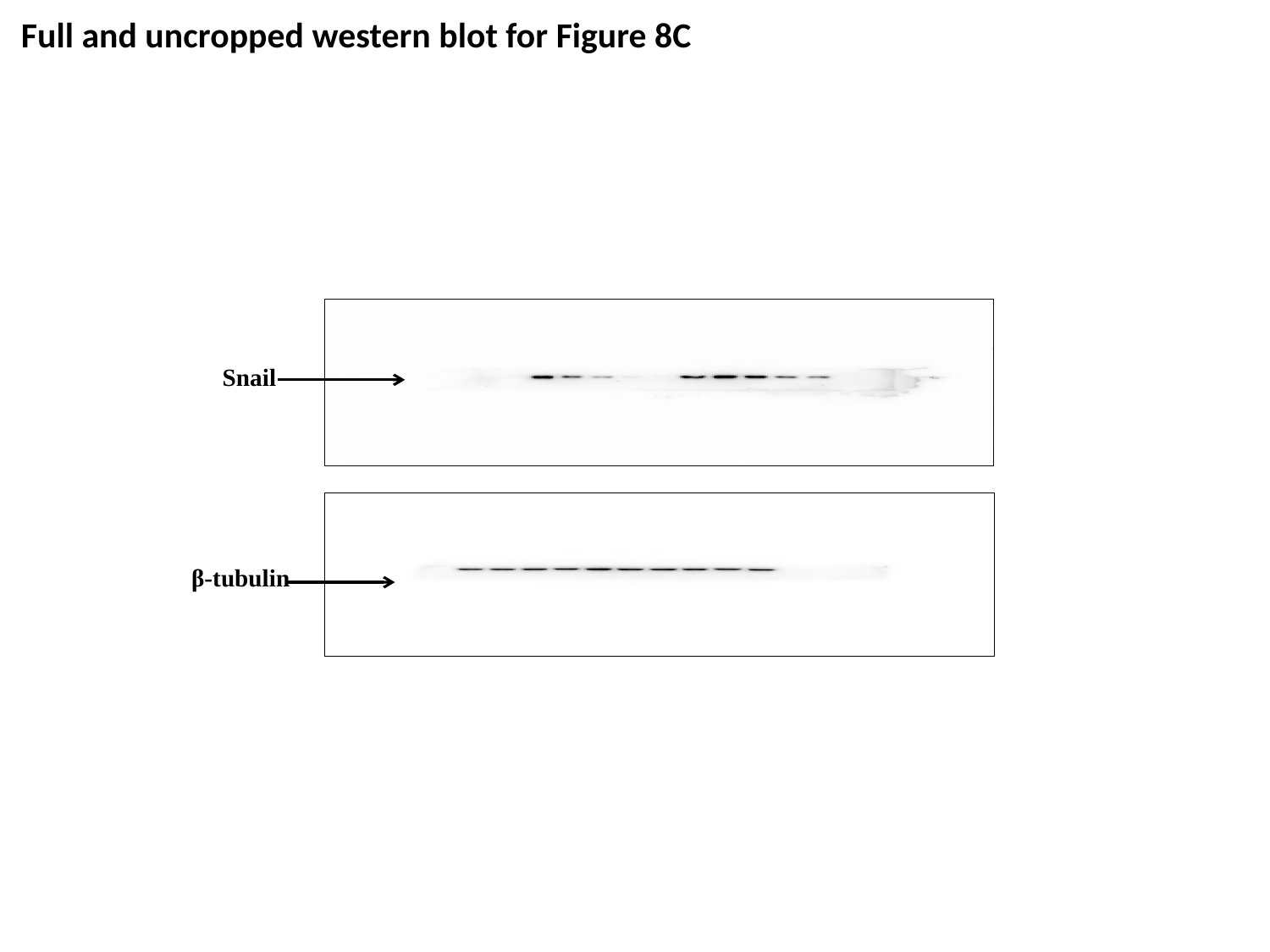

Full and uncropped western blot for Figure 8C
Snail
β-tubulin

## Slide 24
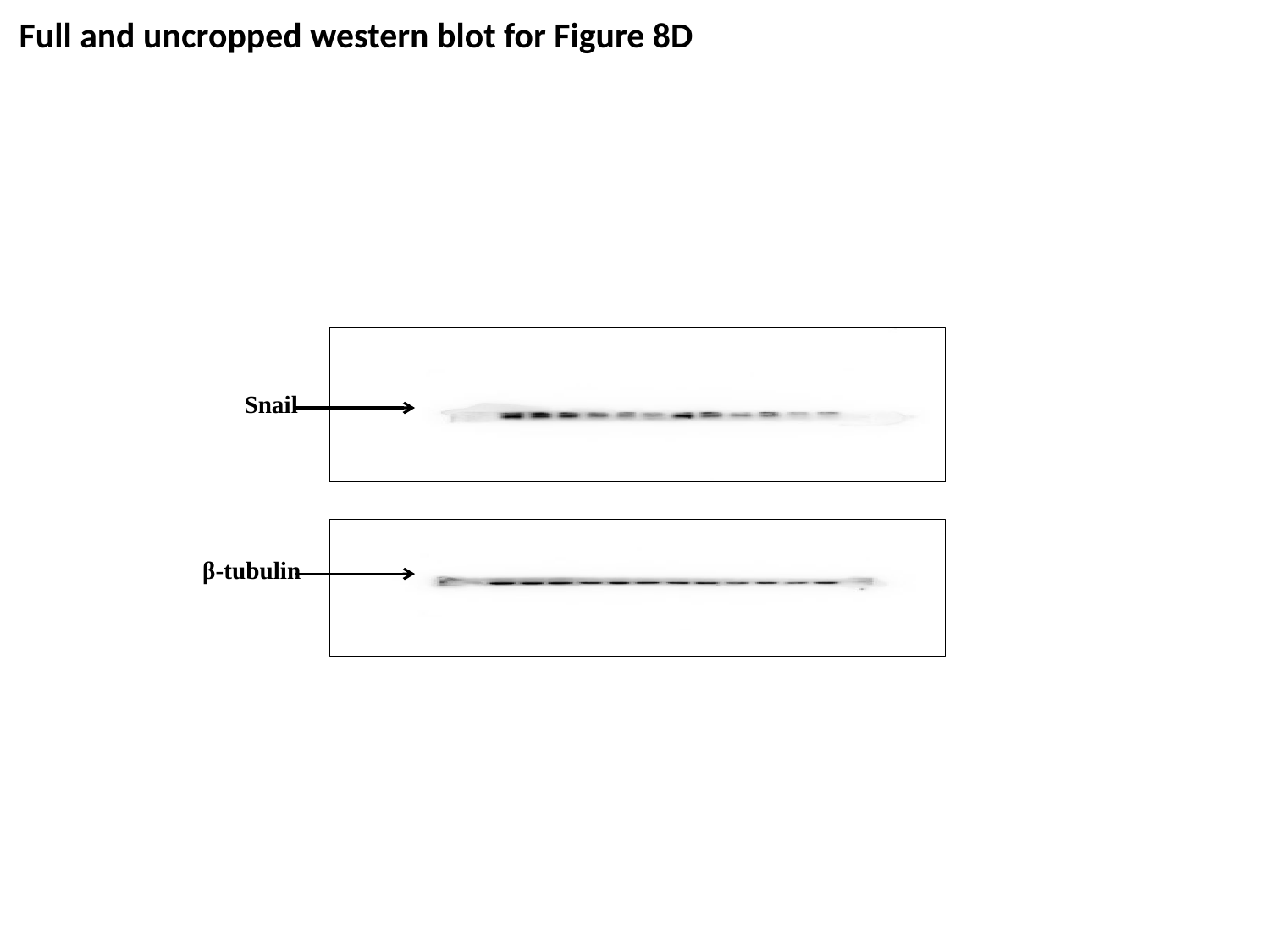

Full and uncropped western blot for Figure 8D
Snail
β-tubulin

## Slide 25
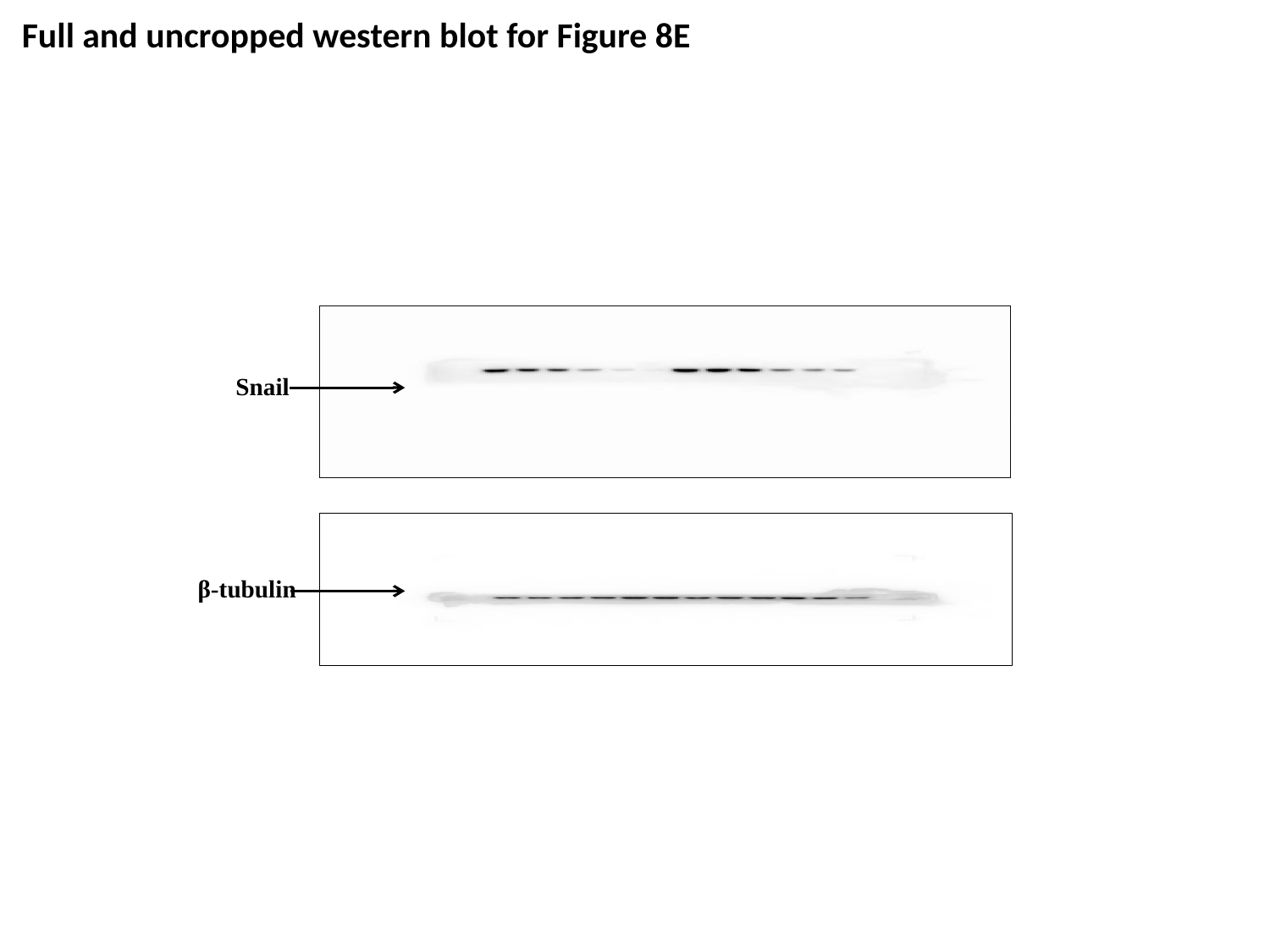

Full and uncropped western blot for Figure 8E
Snail
β-tubulin

## Slide 26
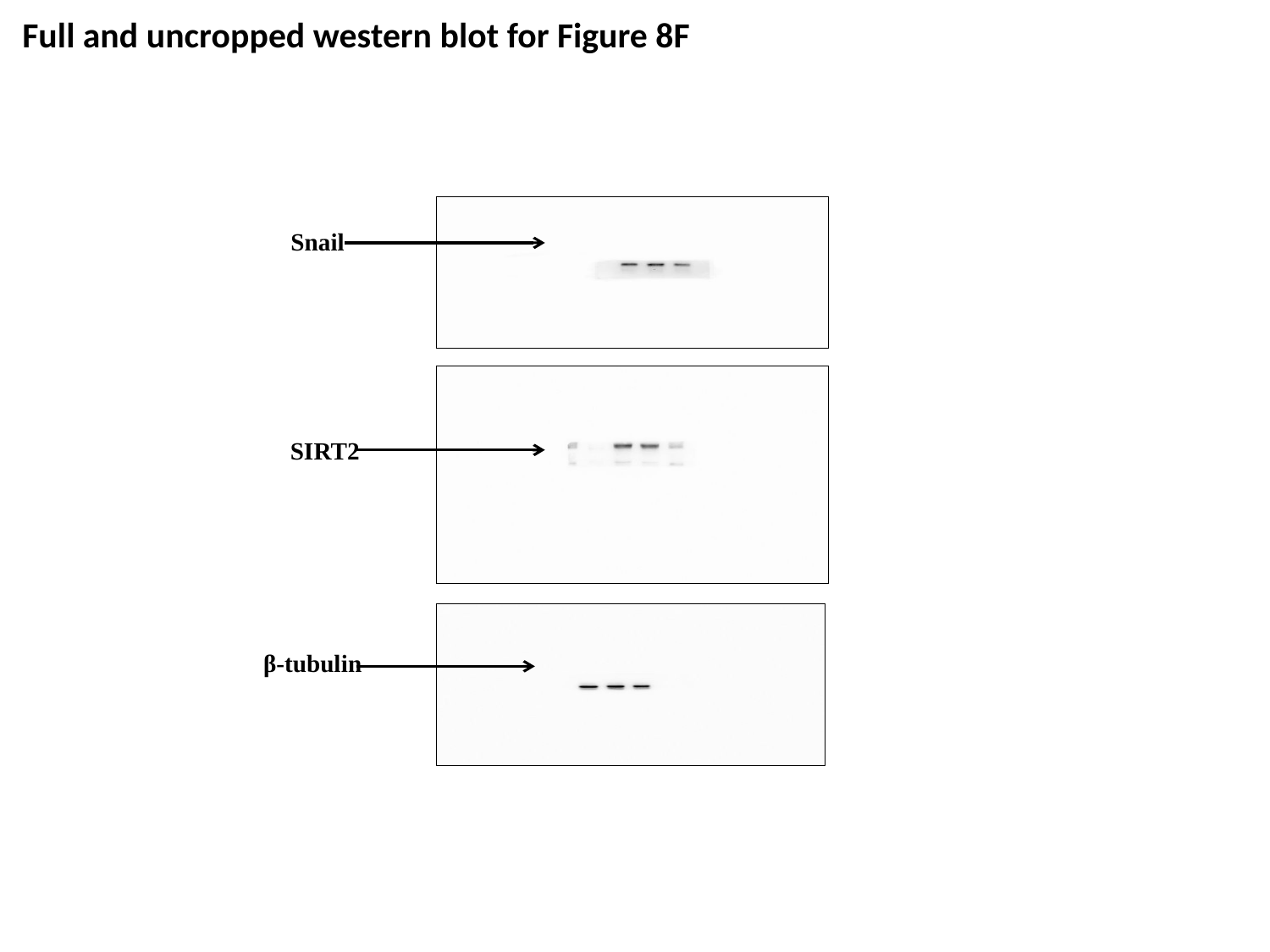

Full and uncropped western blot for Figure 8F
Snail
SIRT2
β-tubulin

## Slide 27
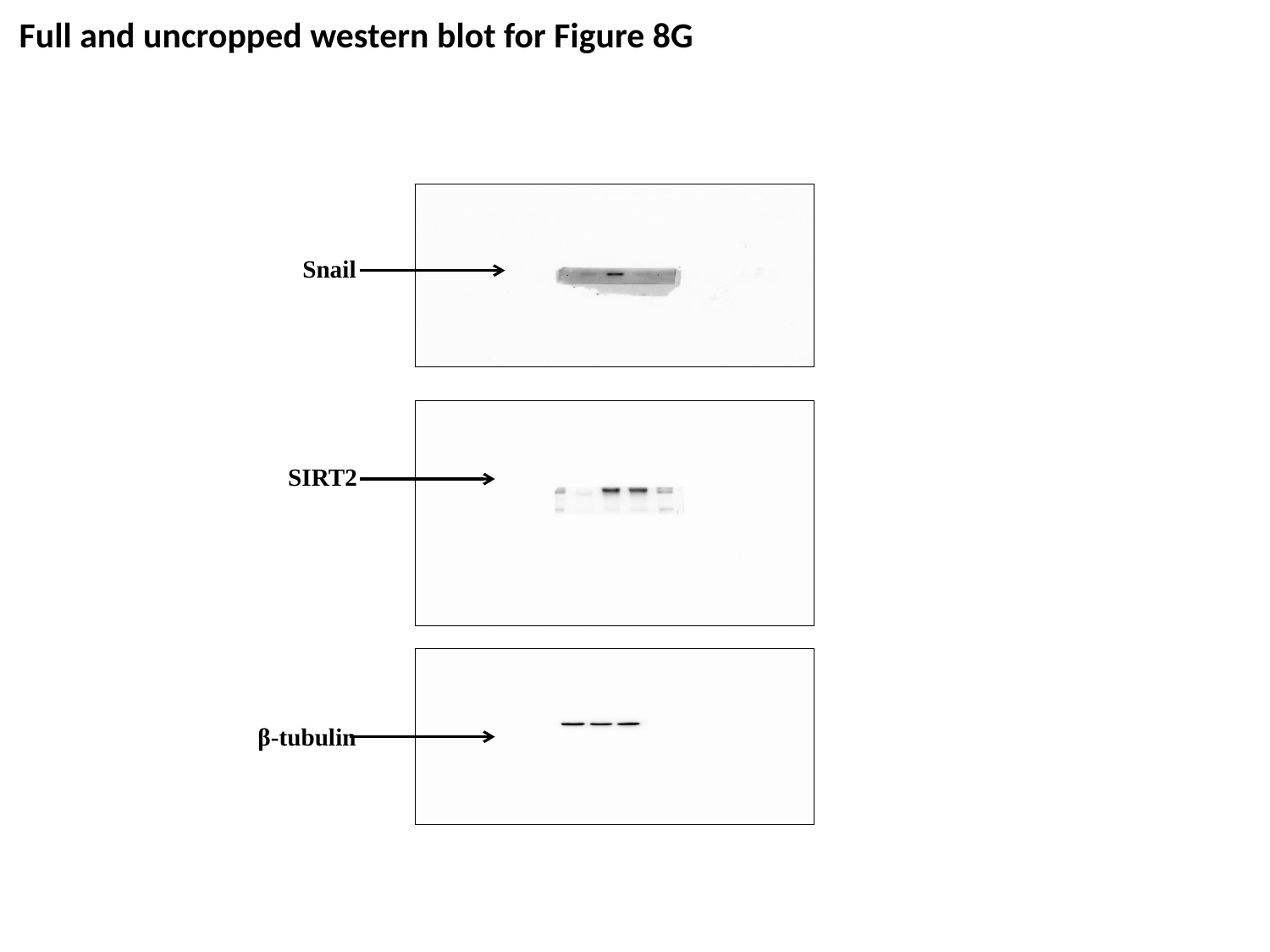

Full and uncropped western blot for Figure 8G
Snail
SIRT2
β-tubulin
